# New Ansamycins from the Deep-Sea-Derived Bacterium *Ochrobactrum* sp. OUCMDZ-2164

**DOI:** 10.3390/md16080282

**Published:** 2018-08-15

**Authors:** Yaqin Fan, Cong Wang, Liping Wang, Arthit Chairoungdua, Pawinee Piyachaturawat, Peng Fu, Weiming Zhu

**Affiliations:** 1 Key Laboratory of Marine Drugs, Ministry of Education of China, School of Medicine and Pharmacy, Ocean University of China, Qingdao 266003, China; fanyaqin.826@163.com (Y.F.); wangcong123206@163.com (C.W.); 2 Laboratory for Marine Drugs and Bioproducts, Qingdao National Laboratory for Marine Science and Technology, Qingdao 266003, China; 3 Guangxi Key Laboratory of Chemistry and Engineering of Forest Products, School of Chemistry and Chemical Engineering, Guangxi University for Nationalities, Nanning 530006, China; 4 State Key Laboratory of Functions and Applications of Medicinal Plants, Guizhou Medical University, Guiyang 550014, China; lipingw2006@163.com; 5 Department of Physiology, Faculty of Science, Mahidol University, Bangkok 10400, Thailand; arthit.chi@mahidol.ac.th (A.C.); pawinee.pia@mahidol.ac.th (P.P.)

**Keywords:** deep-sea-derived bacterium, *Ochrobactrum* sp., ansamycins, trienomycins, cytotoxic activity

## Abstract

Two new ansamycins, trienomycins H (**1**) and I (**2**), together with the known trienomycinol (**3**), were isolated from the fermentation broth of the deep-sea-derived bacterium *Ochrobactrum* sp. OUCMDZ-2164. Their structures, including their absolute configurations, were elucidated based on spectroscopic analyses, ECD spectra, and Marfey’s method. Compound **1** exhibited cytotoxic effects on A549 and K562 cell lines with IC_50_ values of 15 and 23 μM, respectively.

## 1. Introduction

During the last two decades, increasingly more natural product (NP) chemists have set their sights on the sea because of the abundance of biological diversity of marine life. Numerous NPs with novel chemical skeletons and intriguing biological activities have been isolated from the marine organisms, including plants, animals, microorganisms, and so on [1,2,3]. Marine-derived microorganisms, as a huge resource for the discovery of active NPs, have attracted widespread attention [4,5,6,7,8,9,10,11]. Researchers tried to obtain new species from extreme environments, and the deep sea has become an important target [12,13,14,15], which was found to be a habitat with a diversity of species, especially microorganisms. The deep-sea-derived microorganisms have a great potential to produce new active compounds because of the special survival conditions [16,17,18,19]. For example, ammosamides A and B, produced by a deep-sea sediment-derived *Streptomyces* strain (1618 m), showed potent cytotoxicity against the HCT-116 cell line [20].

Ansamycins are a class of bacterial macrocyclic polyketides mainly produced by terrestrial sample-derived *Streptomyces* and *Bacillus* species, and seldom by marine ones [21,22]. Examples include rifamycin, the first ansamycin from a terrestrial soil-derived *Streptomyces* sp. [23]; geldanamycin, the first benzoquinoid ansamycin, from the terrestrial soil-derived *Streptomyces hygroscopicus* UC-5208 [24]; and ansalactam A, from a marine-derived *Streptomyces* sp. CHN-189 [25]. Ansamycins exhibit a broad range of bioactivities, such as antimicrobial [26], antitumor [27], and antiviral [28] activities. The macrocyclic system composed of an aromatic moiety embedded in an alicycle was a distinguishing feature of this class of compounds, attracting much attention from biosynthetic and chemical synthesis researchers [22,29,30]. Usually, ansamycins are classified as two types based on the aromatic moiety, which are, naphthalenic ones and benzenic ones. Naphthalenic ansamycins represented by rifamycin displayed strong antimicrobial activities, while some benzenic ansamycins showed strong anti-tumor activities [31]. For example, 17-allylamino-17-demethoxy geldanamycin (17-AAG) [32], is in phase II clinical trials for the treatment of metastatic melanoma.

**Figure 1:**
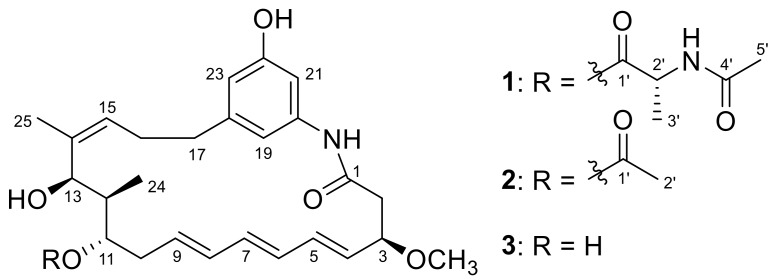
Structures **1**–**3** from *Ochrobactrum* sp. OUCMDZ-2164.

As part of our studies to search for new bioactive NPs from deep-sea-derived bacteria [33,34,35], we found that the extract of the fermentation broth of a deep-sea (2000 m) water-derived *Ochrobactrum* sp. OUCMDZ-2164 showed cytotoxic effect on the A549 (83% inhibition) and K562 (70% inhibition) cell line at 0.1 mg/mL. Additionally, HPLC profiling of these extracts indicated the presence of secondary metabolites with UV absorptions consistent with the ansamycins (λ_max_ 214 and 272 nm). Chemical investigation led to the isolation of two new benzenic ansamycins, trienomycins H (**1**) and I (**2**), along with the known trienomycinol (**3**) [36,37] (Figure 1). The trienomycins A–F [29,38,39] possess different *N*-acyl substituted alanine ester moieties attached to C-11 of the ansamycin-like ring, such as cyclohexanecarboxyl, 3-methylbutanoyl, (*S*)-2-methylbutanoyl, 1-cyclohexene-1-carboxyl, 4-methylpentanoyl, (*E*)-3-pentenoyl, and (*E*)-2-methyl-2-butenoyl, respectively, while the trienomycin G [21] is the isomer of trienomycin A by interchanging the position of 11- and 13- substituents. Trienomycin H (**1**) possesses a *N*-acetylalanine ester moiety at C-11, and trienomycin I (**2**) possesses an acetoxy group. Compound **1** showed selective cytotoxicity against human lung carcinoma cell line (A549) and human leukemia cell line (K562) with IC_50_ values of 15 and 23 μM, respectively. This is the second example of ansamycins from the deep sea-derived bacteria [33].

## 2. Results and Discussion

**Figure 2:**
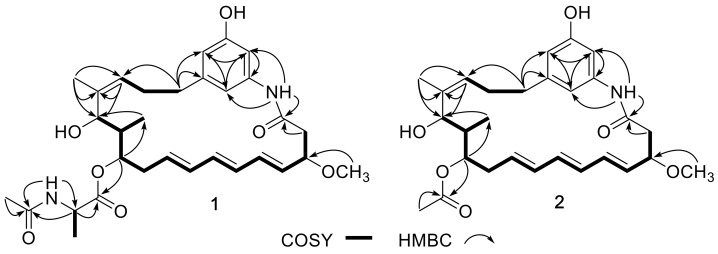
Key COSY and HMBC correlations of compounds **1** and **2**.

Compound **1** was obtained as a yellow oil. Its molecular formula was determined as C_31_H_42_N_2_O_7_ according to the HRESIMS (high resolution electrospray ionization mass spectroscopy) peak at *m*/*z* 555.3058 [M + H]^+^ (calcd. for C_31_H_43_N_2_O_7_, 555.3065) (Figure S1), indicating 12 degrees of unsaturation. The UV spectrum of **1** showed similar absorptions with trienomycins at λ_max_ 214, 254, 258, 272, and 284 nm [36]. The ^1^H NMR spectrum (Table 1, Figure S2) showed three signals at *δ*_H_ 6.30 (s), 6.43 (s), and 6.85 (s), as a result of a 1,3,5-trisubstituted benzene ring system. The conjugated triene signals at δ_H_ 5.54−5.58 (2H, m, H-4 and H-9) and *δ*_H_ 6.05−6.15 (4H, m, H-5, H-6, H-7, and H-8) were also observed in the ^1^H NMR spectrum (Table 1). The ^13^C NMR revealed 31 carbon signals, which were classified by DEPT (distortionless enhancement by polarization transfer) and HSQC (heteronuclear single-quantum correlation) spectra as five methyl carbons (including a methoxy group), four methylene carbons, fifteen methine carbons (including 10 olefinic methine carbons), and seven quaternary carbons (including three carbonyls) (Table 1, Figures S3–S5). Analysis of ^1^H and ^13^C NMR data revealed that compound **1** shared the same ansamycin-like ring system as that of trienomycinol (**3**) [36], which was supported by the COSY (correlation spectroscopy) correlations of H-2/H-3/H-4/H-5/H-6/H-7/H-8/H-9/H-10/H-11/H-12/H-13, H-15/H-16/H-17, and H-12/H-24 (Figure S6), together with the key HMBC (heteronuclear multiple bond correlation) correlations of 3-OCH_3_ to C-3, H-2 to C-1, H-24 to C-13, H-15 to C-13/C-14, H-17 to C-18/C-19/C-23, H-19 to C-21, H-23 to C-19, H-21 to C-19/C-23, and 20-NH to C-1/C-19/C-21 (Figure 2 and Figure S7). Careful comparison of the NMR data between **1** and **3** revealed observation of the signals of an *N*-acetylalanine in compound **1**, which was confirmed by the COSY correlation of H-2′/H-3′, and the HMBC correlations of H-2′ to C-1′/C-4′, 2′-NH to C-2′/C-4′, and H-5′ to C-4′ (Figure 2). The connection of ansamycin-like ring moiety and *N*-acetylalanine unit was determined based on the HMBC correlation of H-11 to C-1′ (Figure 2). To determine the configuration of compound **1**, the hydrolysis was carried out under acidic condition. The water-insoluble hydrolysate was identified as trienomycinol (**3**) by the same HPLC behaviors (Figure S15), ESIMS (Figures S16 and S17), ^1^H NMR (Figure S18), and specific rotation (**1**: $[\alpha {]}_{D}^{25}$ +52.1 (*c* 0.1, MeOH); **3**: [α${]}_{D}^{25}$ +62.8 (*c* 0.1, MeOH)).

**Figure 3:**
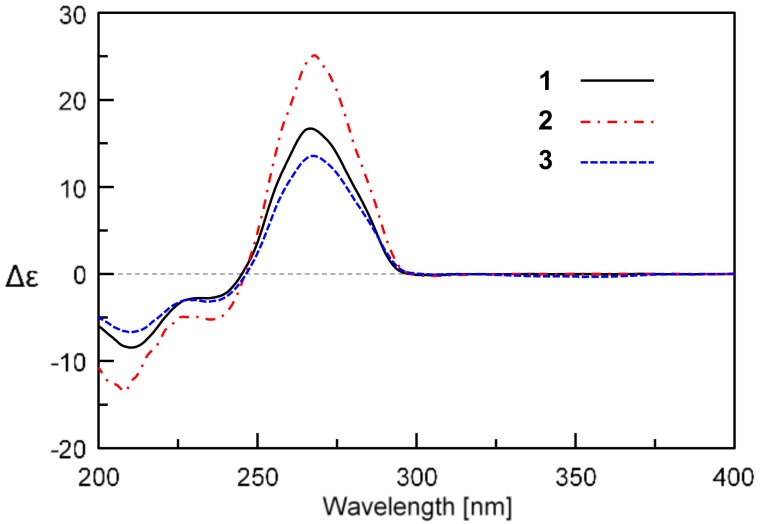
ECD curves of compounds **1**−**3**.

The similar ECD (electronic circular dichroism) Cotton effects of **1** to those of **3** further supported the absolute configuration of the ansa ring system (Figure 3). The absolute configuration of alanine unit was determined as d- by Marfey’s method [40]. The 1-fluoro-2,4-dinitrophenyl-5-l-alanine amide (FDAA) derivative of the acidic hydrolysate of **1** gave the same HPLC retention time as that of authentic d-Ala FDAA derivative (Figure S14). Thus, compound **1** that we named trienomycin H was identified as 11-*O*-trienomycinol *N*-acetyl-d-alaninoate.

The molecular formula of trienomycin I (**2**) was assigned to be C_28_H_37_NO_6_ based on the HRESIMS peak at *m*/*z* 484.2697 [M + H]^+^ and 506.2513 [M + Na]^+^ (Figure S8). The ^1^H and ^13^C NMR data (Table 1, Figures S9–S11) and 2D NMR pattern (Figures S12 and S13) of **2** were very similar to those of compound **3** except for two additional carbon signals (*δ*_C_ 169.8, 20.7) and a singlet methyl signal (*δ*_H_ 2.00, s) corresponding to an acetyl group. Analysis of the molecular formula and the HMBC correlations of H-2′ to C-1′ and H-11 to C-1′ (Figure 2) revealed that compound **2** was an acetate of **3** at 11-OH. The water-insoluble part (ethyl acetate (EtOAc) extract) of the acidic hydrolysate of **2** was determined to be **3** based on ^1^H NMR (Figure S18), HPLC analysis (Figure S19), ESIMS (Figure S20), and specific rotation ($[\alpha {]}_{D}^{25}$+47.8 (*c* 0.1, MeOH)). Thus, the structure **2** was unambiguously determined as 11-*O*-trienomycinol acetate.

Compounds **1**−**3** were evaluated for cytotoxicity against the human leukemia cell line (K562), human breast adenocarcinoma cell line (MCF-7), and human lung carcinoma cell line (A549). Only compound **1** showed selectively cytotoxic activity against A549 and K562 cell lines with the IC_50_ values of 15 and 23 μM, respectively, while compounds **2** and **3** did not show cytotoxic activities against three tumor cell lines. The results indicated that the *N*-acylalanine ester moiety is required for the cytotoxicity, in accordance with the literature data [36]. 

## 3. Experimental Section

### 3.1. General Experimental Procedures

Optical rotations were measured with a JASCO P-1020 digital polarimeter (JASCO Corporation, Tokyo, Japan) equipped with a halogen lamp (589 nm). UV spectra were recorded on a Beckman DU 640 spectrophotometer (Beckman Coulter Inc., Brea, CA, USA). ECD spectra were measured on JASCO J-815 spectrometer (JASCO Corporation, Tokyo, Japan). IR spectra were obtained on a Nicolet NEXUS 470 spectrophotometer (Thermo Nicolet Corporation, Madison, WI, USA) as KBr discs. ^1^H and ^13^C NMR, DEPT NMR, and 2D NMR spectra were recorded on a Bruker Avance 600 spectrometer (Bruker, Fällanden, Switzerland). Chemical shifts were referenced to the corresponding solvent residual signal (*δ*_H_ 2.50 and *δ*_C_ 39.52 in DMSO-*d*_6_). HRESIMS spectra were recorded using the Q-TOF ULTIMA GLOBAL GAA076 LC mass spectrometer (Waters Asia Ltd., Singapore). Semi-preparative HPLC was performed using an ODS column (YMC-pack ODS-A, 10 × 250 mm, 5 μm, 4.0 mL/min, Kyoto, Japan). Thin-layer chromatography (TLC) was performed on plates precoated with silica gel GF_254_ (10−40 μm). Silica gel (200−300 mesh, Qingdao Marine Chemical Factory, Qingdao, China) and Sephadex LH-20 (Amersham Biosciences, Uppsala Sweden) were used for column chromatography (CC). Vacuum-liquid chromatography (VLC) was carried out over silica gel H (Qingdao Marine Chemical Factory, Qingdao, China).

### 3.2. Collection and Phylogenetic Analysis

The bacterial strain OUCMDZ-2164 was isolated from the deep-sea water sample collected from the South China Sea (2000 m depth) in April 2012. The sea water sample was collected by a conductance temperature depth (CTD) device (Qingdao, China). The sea water (3.0 mL) was deposited on agar plate (3 g/L beef extract, 20 g/L glucose, 10 g/L yeast extract, 10 g/L soluble starch, 10 g/L peptone, 2 g/L CaCO_3_, 0.5 g/L KH_2_PO_4_, and 0.5 g/L MgSO_4_, in seawater) containing nystatin (100 μg/mL) as a fungi inhibitor and incubated at 28 °C for 8 days, until a single colony appeared. The single colony was transferred into another agar plate. It was characterized as *Ochrobactrum* sp. according to its 16S rRNA gene sequences (GenBank accession No. KX394628).

### 3.3. Cultivation and Extraction

Bacterium OUCMDZ-2164 was cultivated in 500 Erlenmeyer flasks (500 mL, Chengdu, China) each containing 150 mL of a seawater-based medium (3 g/L beef extract, 20 g/L glucose, 10 g/L yeast extract, 10 g/L soluble starch, 10 g/L peptone, 2 g/L CaCO_3_, 0.5 g/L KH_2_PO_4_, and 0.5 g/L MgSO_4_, pH 7.5−8.0) and shaken for nine days (28 ± 0.5 °C, 180 rpm). The whole broth was extracted three times with ethyl acetate (EtOAc) (60 L each). The EtOAc extract was concentrated in vacuo to give 22.0 g of crude extract.

### 3.4. Purification

The crude extract of strain OUCMDZ-2164 (22.0 g) was fractionated into 11 fractions (Fr.1−Fr.11) by flash column chromatography on silica gel, eluting with a stepwise gradient of petroleum ether/CH_2_Cl_2_ (1:1 and 0:1) followed by CH_2_Cl_2_/MeOH (100−0%). Fr.9 (1.69 g) was separated into six fractions (Fr.9.1−Fr.9.6) by Sephadex LH-20, eluting with CH_2_Cl_2_/MeOH (1:1). Fr.9.2 (563 mg) was purified by semi-preparative HPLC on ODS column eluting with 55% MeOH/H_2_O to yield compounds **2** (3.0 mg, *t*_R_ 11.0 min) and **1** (10.0 mg, *t*_R_ 15.2 min). Fr.10 (925 mg) was separated into four fractions (Fr.10.1−Fr.10.4) by Sephadex LH-20, eluting with CH_2_Cl_2_/MeOH (1:1). Fr.10.2 (112 mg) was further purified by semi-preparative HPLC on ODS column eluting with 65% MeOH/H_2_O to yield trienomycinol (**3**) (8.9 mg, *t*_R_ 6.3 min).

**Trienomycin H (1)**: Yellow oil; $[\alpha {]}_{D}^{25}$ +64.1 (*c* 0.2, MeOH); UV (MeOH) λ_max_ (log ε): 214 (2.25), 254 (1.78), 258 (1.83), 272 (2.01), 284 (1.69) nm; ECD (0.0011 *M*, MeOH) λ_max_ (Δε) 211 (−8.4), 234 (−2.8), 267 (+16.7) nm; IR (KBr) ν_max_ 3430, 2931, 2840, 1738, 1659, 1625, 1545, 1451, 1380, 1100, 1000 cm^−1^; ^1^H and ^13^C NMR data, see Table 1; HRESIMS *m*/*z* 555.3058 [M + H]^+^ (calcd. for C_31_H_43_N_2_O_7_, 555.3065).

**Trienomycin I (2)**: Yellow oil; $[\alpha {]}_{D}^{25}$ +67.9 (*c* 0.2, MeOH); UV (MeOH) λ_max_ (log ε): 212 (3.16), 220 (2.92), 252 (2.88), 260 (3.00), 272 (3.35), 284 (2.77) nm; ECD (0.0012 *M*, MeOH) λ_max_ (Δε) 209 (−13.4), 235 (−5.2), 268 (+25.1) nm; IR (KBr) ν_max_ 3392, 2932, 2860, 1730, 1660, 1622, 1549, 1443, 1383, 1080, 1000 cm^−1^; ^1^H and ^13^C NMR data, see Table 1; HRESIMS *m*/*z* 484.2697 [M + H]^+^ (calcd. for C_28_H_38_NO_6_, 484.2694); *m*/*z* 506.2513 [M + Na]^+^ (calcd. for C_28_H_37_NO_6_Na, 506.2513).

### 3.5. Determination of the Absolute Configurations of ***1*** and ***2***

A solution of compound **1** (2.0 mg) in 6.0 M HCl (1.5 mL) was placed in an ampoule flask. It was then sealed and heated at 105 °C for 17 h. The reaction mixture was diluted with H_2_O (1 mL) and extracted with EtOAc (3 × 15 mL). The organic layer was combined and concentrated to give the water-insoluble hydrolysate (1.0 mg) that was identified as trienomycinol (**3**) by Co-HPLC (Figure S15), ESIMS (Figure S16), ^1^H NMR (Figure S18), and specific rotation. The water solution, after being evaporated to dryness and re-dissolved in H_2_O (250 μL), was used to determination of alanine configuration by Marfey’s method. Then, 50 μL of the acid hydrolysates solution was placed in a 1 mL reaction vial and treated with 1% solution of FDAA (200 μL) in acetone followed by 1.0 M NaHCO_3_ (40 μL). The reaction mixture was heated at 45 °C for 1 h, and then acidified with 2.0 *M* HCl (20 μL). In a similar fashion, standard d- and l-Ala were derivatized separately. The derivatives of the hydrolysates and standard amino acids were subjected to HPLC analysis (YMC-pack ODS-A column, Kyoto, Japan); 5 μm, 4.6 × 250 mm; 1.0 mL/min) at 30 °C using the following gradient program: solvent A, water + 0.2% TFA; solvent B, MeCN; linear gradient: 0 min 25% B, 40 min 60% B, 45 min 100% B; UV detection at λ 340 nm. The retention times for the FDAA derivatives of the hydrolysate of **1**, standard d-Ala, and l-Ala were 14.7, 14.7, and 12.5 min, respectively (Figure S14). By the same procedure, the water-insoluble hydrolysate (0.9 mg) of compound **2** was obtained from the sealed reaction of compound **2** (2.0 mg) with 6.0 M HCl (1.5 mL), which was identified as **3** by Co-HPLC (Figure S19), ESIMS (Figure S20), ^1^H NMR (Figure S18), and specific rotation.

### 3.6. Cytotoxic Assays

Cytotoxicity was assayed by the MTT [41] and CCK-8 [42,43] methods. In the MTT assay, A549 or MCF-7 cell line was grown in RPMI-1640 supplemented with 10% FBS under a humidified atmosphere of 5% CO_2_ and 95% air at 37 °C, respectively. Cell suspension, 100 μL, at a density of 3 × 10^4^ cell/mL was plated in 96-well microtiter plates, allowed to attach overnight, and then exposed to varying concentrations (10^−5^−10^−12^ M) of compounds for 72 h. The MTT solution (20 μL, 5 mg/mL in IPMI-1640 medium) was then added to each well and incubated for 4 h. Old medium containing MTT was then gently replaced by DMSO and pipetted to dissolve any formazan crystals formed. Absorbance was then determined on a Spectra Max Plus plate reader at 570 nm. In the CCK-8 assay, K562 cell line was grown in RPMI-1640 supplemented with 10% FBS under a humidified atmosphere of 5% CO_2_ and 95% air at 37 °C. Cell suspension, 100 μL, at a density of 5 × 10^4^ cell/mL was plated in 96-well microtiter plates and then exposed to varying concentrations (10^−5^−10^−12^ M) of compounds after cultivation for 24 h. Three days later, 10 μL of CCK-8 solution was added 4 h before detection. Then, the absorbance (450 nm) was measured, and the growth rates of cells were computed. Adriamycin was used as the positive control with the IC_50_ values of 1.00, 0.63, and 0.73 for the cell lines MCF-7, A549, and K562, respectively.

## 4. Conclusions

The present study revealed two new ansamycins, trienomycins H and I (**1** and **2**), from a deep-sea-derived bacterial strain, *Ochrobactrum* sp. OUCMDZ-2164. As the first example of ansamycin reported from a French soil-derived bacterium in 1950s [23], this is the first report of new ansamycins from the deep sea-derived bacteria. Trienomycin H (**1**) exhibited selectively cytotoxic effects on A549 and K562 cell lines with the IC_50_ values of 15 and 23 μM, respectively. Combined with the data of trienomycins A–E against HeLa and P388 cell lines [36], our results supported that *N*-acylalanine ester moiety at C-11 is the key group responsible for the cytotoxic effects of trienomycins against tumor cell lines.

## Figures and Tables

**Table 1:** ^1^H (600 MHz) and ^13^C (150 MHz) NMR data for compounds **1** and **2** in DMSO-*d*_6_.

No.	1		2	
	*δ*_C_	*δ*_H_, mult. (*J* in Hz)	*δ*_C_	*δ*_H_, mult. (*J* in Hz)
1	167.7, C		167.6, C	
2	43.3, CH_2_	2.63, dd (11.6, 3.8); 2.34, m	43.3, CH_2_	2.68, dd (12.0, 4.5); 2.34, m
3	79.8, CH	3.98, m	79.8, CH	3.99, ddd (10.9, 8.4, 4.6)
3-OCH_3_	55.7, CH_3_	3.20, s	55.6, CH_3_	3.21, s
4	131.9, CH	5.55, m *^a^*	131.5, CH	5.53, dd (15.0, 8.3)
5	133.0, CH	6.06, m *^a^*	132.1, CH	6.10, m *^a^*
6	129.8, CH	6.06, m *^a^*	129.1, CH	6.09, m *^a^*
7	133.2, CH	6.12, m *^a^*	133.7, CH	6.08, m *^a^*
8	133.1, CH	6.07, m *^a^*	133.2, CH	6.05, m *^a^*
9	129.5, CH	5.54, m *^a^*	131.4, CH	5.72, ddd (14.7, 10.1, 4.6)
10	32.3, CH_2_	2.42, m; 2.21, m	35.5, CH_2_	2.44, m; 2.33, m
11	74.1, CH	4.66, brs	72.3, CH	5.62, d (4.6)
12	38.7, CH	1.74, m	39.1, CH	1.71, m
13	67.1, CH	4.47, brs	69.4, CH	4.72, brs
14	139.5, C		139.5, C	
15	123.5, CH	5.10, brs	125.6, CH	5.19, m
16	28.8, CH_2_	2.23, m; 1.80, m	29.0, CH_2_	2.20, m; 2.18, m
17	35.8, CH_2_	2.44, m; 2.34, m	36.2, CH_2_	2.44, m; 2.33, m
18	142.9, C		142.7, C	
19	111.2, CH	6.30, s	111.0, CH	6.30, s
20	139.5, C		139.5, C	
21	105.3, CH	6.85, s	105.2, CH	6.82, s
22	157.3, C		157.3, C	
23	111.2, CH	6.43, s	111.1, CH	6.47, s
24	9.7, CH_3_	0.79, d (6.5)	10.2, CH_3_	0.78, d (6.0)
25	20.6, CH_3_	1.66, s	20.3, CH_3_	1.56, s
1′	171.9, C		169.8, C	
2′	48.2, CH	4.19, dq (6.2, 7.2)	20.7, CH_3_	2.00, s
3′	16.9, CH_3_	1.24, d (7.2)		
4′	169.6, C			
5′	22.2, CH_3_	1.81, s		
20-NH		9.54, s		9.52, s
2′-NH		8.34, d (6.2)		

*^a^* Overlapped.

## Supplementary Materials

The following are available online at http://www.mdpi.com/1660-3397/16/8/282/s1, Figure S1: HRESIMS spectrum of trienomycin H (1), Figure S2: ^1^H-NMR spectrum of trienomycin H (1) in DMSO-*d*_6_, Figure S3: ^13^C-NMR spectrum of trienomycin H (1) in DMSO-*d*_6_, Figure S4: DEPT spectrum of trienomycin H (1) in DMSO-*d*_6_, Figure S5: HSQC spectrum of trienomycin H (1) in DMSO-*d*_6_, Figure S6: ^1^H-^1^H COSY spectrum of trienomycin H (1) in DMSO-*d*_6_, Figure S7: HMBC spectrum of trienomycin H (1) in DMSO-*d*_6_, Figure S8: HRESIMS spectrum of trienomycin I (2), Figure S9: ^1^H-NMR spectrum of trienomycin I (2) in DMSO-*d*_6_, Figure S10: ^13^C-DEPTQ-NMR spectrum of trienomycin I (2) in DMSO-*d*_6_, Figure S11: HSQC spectrum of trienomycin I (2) in DMSO-*d*_6_, Figure S12: ^1^H-^1^H COSY spectrum of trienomycin I (2) in DMSO-*d*_6_, Figure S13: HMBC spectrum of trienomycin I (2) in DMSO-*d*_6_, Figure S14: The determination of the Ala configuration of 1 by Marfey’s Method, Figure S15: HPLC profiles for the water-insoluble hydrolysate of 1 and 3, Figure S16: ESIMS spectrum for the water-insoluble hydrolysate of 1, Figure S17: ESIMS spectrum of trienomycinol (3), Figure S18: ^1^H NMR spectra of 3 and the water-insoluble hydrolysates of 1 and 2, Figure S19: HPLC profiles for the water-insoluble hydrolysates of 2 and 3, Figure S20: ESIMS spectrum for the water-insoluble hydrolysate of 2, Figure S21: Phylogenetic tree mapping for the *Ochrobactrum* sp. OUCMDZ-2164, The physical properties of trienomycinol (3).
