# Supplementary material for: New Ansamycins from the Deep-Sea-Derived Bacterium Ochrobactrum sp. OUCMDZ-2164
Source: Mar Drugs. 2018 Aug 15;16(8):282. doi: 10.3390/md16080282 (PMC6117703; doi:10.3390/md16080282)
Supplement: Supplementary file 1 [file marinedrugs-16-00282-s001.pdf]

Supplementary Material

New Ansamycins from the Deep-Sea-Derived Bacterium *Ochrobactrum* sp. OUCMDZ-2164

Yaqin Fan [1,2,†], Cong Wang [1,3,†], Liping Wang [4], Arthit Chairoungdua [5], Pawinee Piyachaturawat [5], Peng Fu [1,2*] and Weiming Zhu [1,2,4*]

1 Key Laboratory of Marine Drugs, Ministry of Education of China, School of Medicine and Pharmacy, Ocean University of China, Qingdao 266003, China; fanyaqin.826@163.com (Y.F.); wangcong123206@163.com (C.W.)
2 Laboratory for Marine Drugs and Bioproducts, Qingdao National Laboratory for Marine Science and Technology; Qingdao 266003, China
3 Guangxi Key Laboratory of Chemistry and Engineering of Forest Products, School of Chemistry and Chemical Engineering, Guangxi University for Nationalities, Nanning 530006, China
4 State Key Laboratory of Functions and Applications of Medicinal Plants, Guizhou Medical University, Guiyang 550014, China; lipingw2006@163.com
5 Department of Physiology, Faculty of Science, Mahidol University, Bangkok 10400, Thailand; arthit.chi@mahidol.ac.th (A.C.); pawinee.pia@mahidol.ac.th (P.P.)

* Correspondence: fupeng@ouc.edu.cn (P.F.); weimingzhu@ouc.edu.cn (W.Z.); Tel./Fax: +86-532-8203-1268 (W.Z.)

† These authors contributed equally to this paper.

List of Supplementary Material

Figure S1. HRESIMS spectrum of trienomycin H (**1**)……………………………………………S3
Figure S2. ^{1}H-NMR spectrum of trienomycin H (**1**) in DMSO-d_6…………………………………S4
Figure S3. ^{13}C-NMR spectrum of trienomycin H (**1**) in DMSO-d_6…………………………………S5
Figure S4. DEPT spectrum of trienomycin H (**1**) in DMSO-d_6……………………………………S6
Figure S5. HSQC spectrum of trienomycin H (**1**) in DMSO-d_6……………………………………S7
Figure S6. ^{1}H-^{1}H COSY spectrum of trienomycin H (**1**) in DMSO-d_6……………………………S8
Figure S7. HMBC spectrum of trienomycin H (**1**) in DMSO-d_6……………………………………S9
Figure S8. HRESIMS spectrum of trienomycin I (**2**) ………………………………………………S10
Figure S9. ^{1}H-NMR spectrum of trienomycin I (**2**) in DMSO-d_6…………………………………S11
Figure S10. ^{13}C-DEPTQ-NMR spectrum of trienomycin I (**2**) in DMSO-d_6……………………S12
Figure S11. HSQC spectrum of trienomycin I (**2**) in DMSO-d_6……………………………………S13
Figure S12. ^{1}H-^{1}H COSY spectrum of trienomycin I (**2**) in DMSO-d_6…………………………S14
Figure S13. HMBC spectrum of trienomycin I (**2**) in DMSO-d_6……………………………………S15
Figure S14. The determination of the Ala configuration of **1** by Marfey's Method ……………………S16
Figure S15. HPLC profiles for the water-insoluble hydrolysate of **1** and **3**…………………………S17
Figure S16. ESIMS spectrum for the water-insoluble hydrolysate of **1**……………………………S18
Figure S17. ESIMS spectrum of trienomycinol (**3**) ……………..…….………………………....S19
Figure S18. ^{1}H NMR spectra of **3** and the water-insoluble hydrolysate of **1** and **2**……… ……………S20
Figure S19. HPLC profiles for the water-insoluble hydrolysate of **2** and **3**…………………………S21
Figure S20. ESIMS spectrum for the water-insoluble hydrolysate of **2**……………………………S22
Figure S21. Phylogenetic tree mapping for the *Ochrobactrum* sp. OUCMDZ-2164 …………………S23
The Physical Properties of trienomycinol (**3**) …………………………………………………S24

Figure S1. HRESIMS spectrum of trienomycin H (**1**)

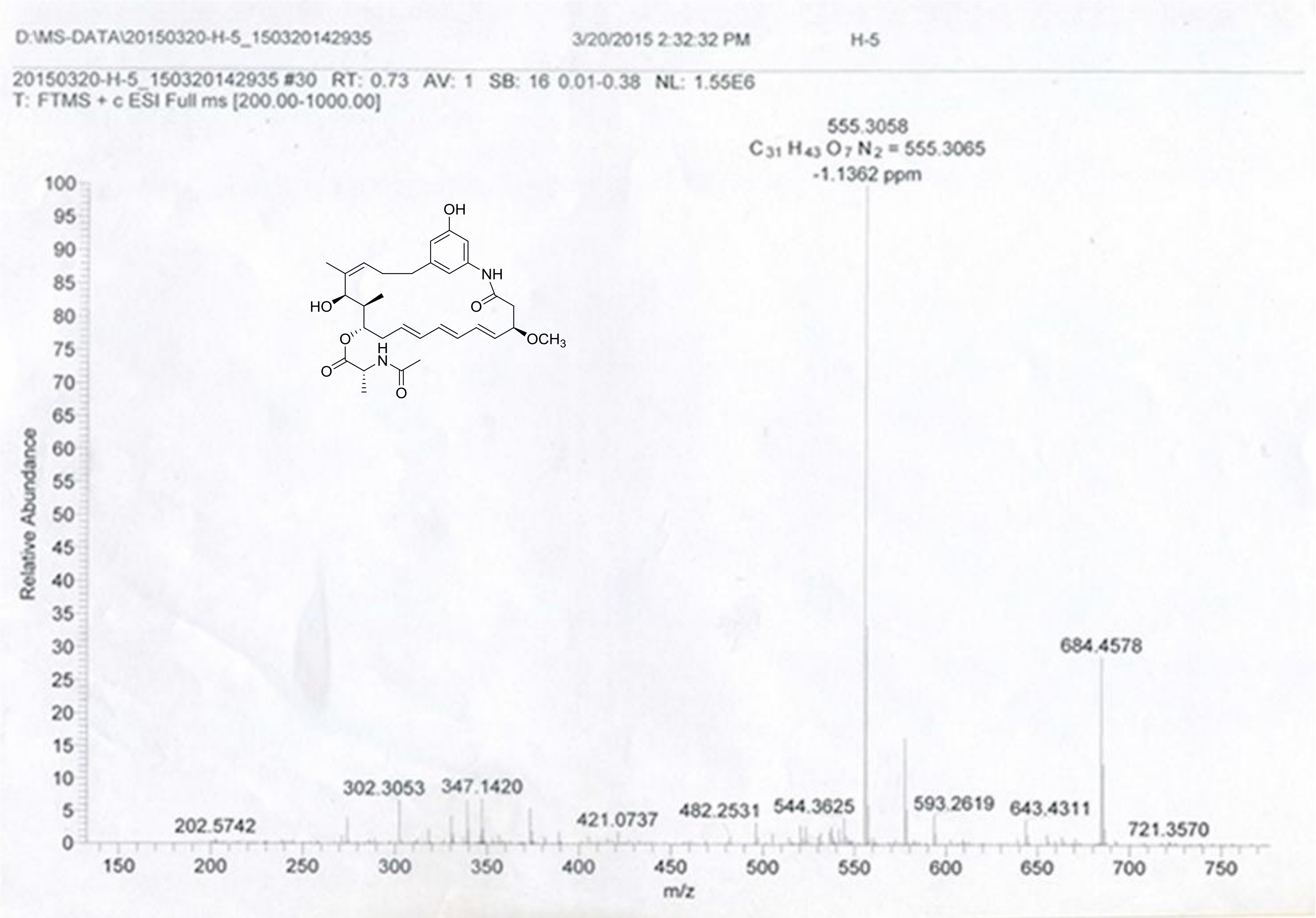

Figure S2. ^{1}H-NMR spectrum of trienomycin H (**1**) in DMSO-d_6

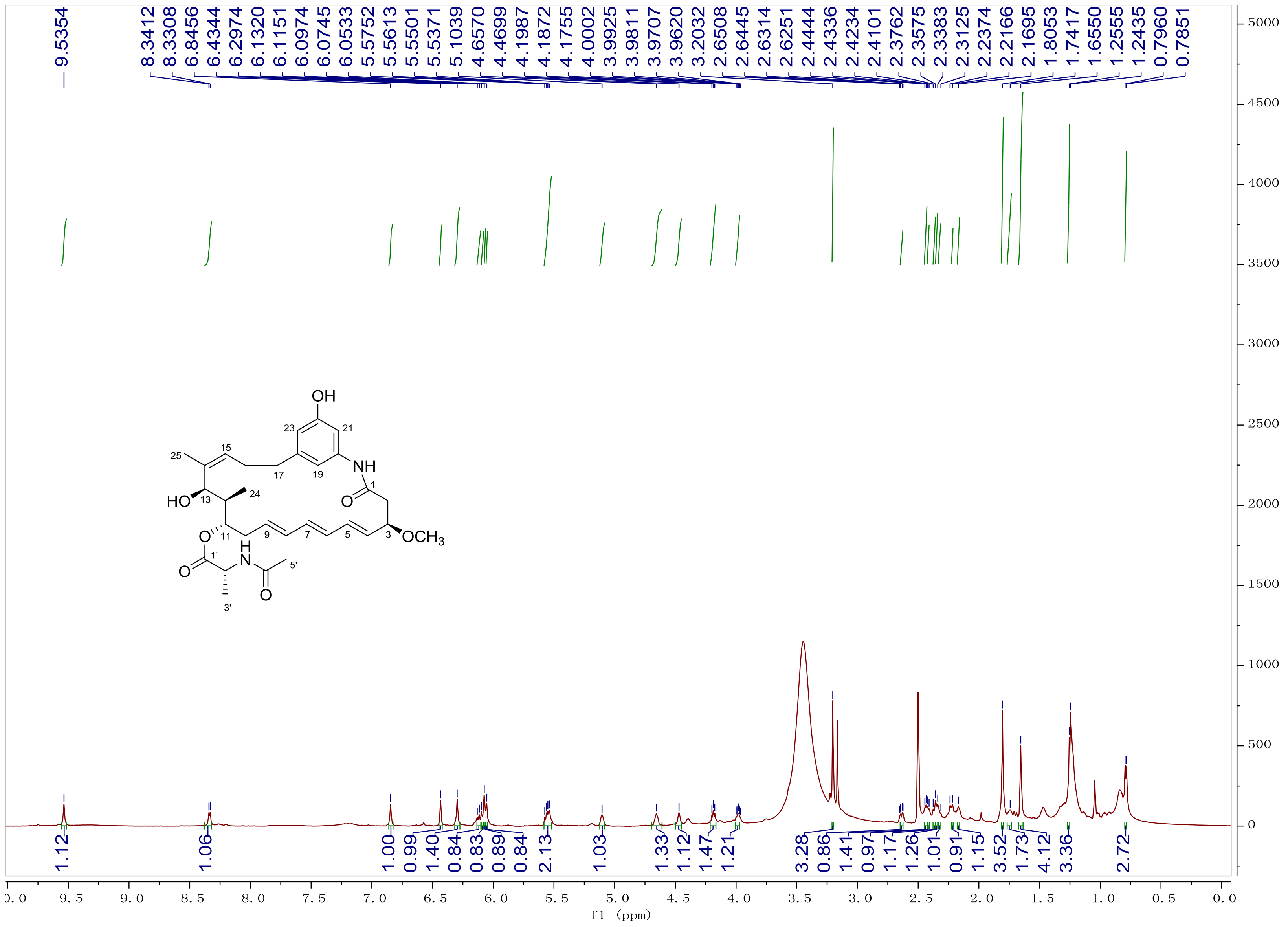

Figure S3. ^{13}C-NMR spectrum of trienomycin H (**1**) in DMSO-d_6

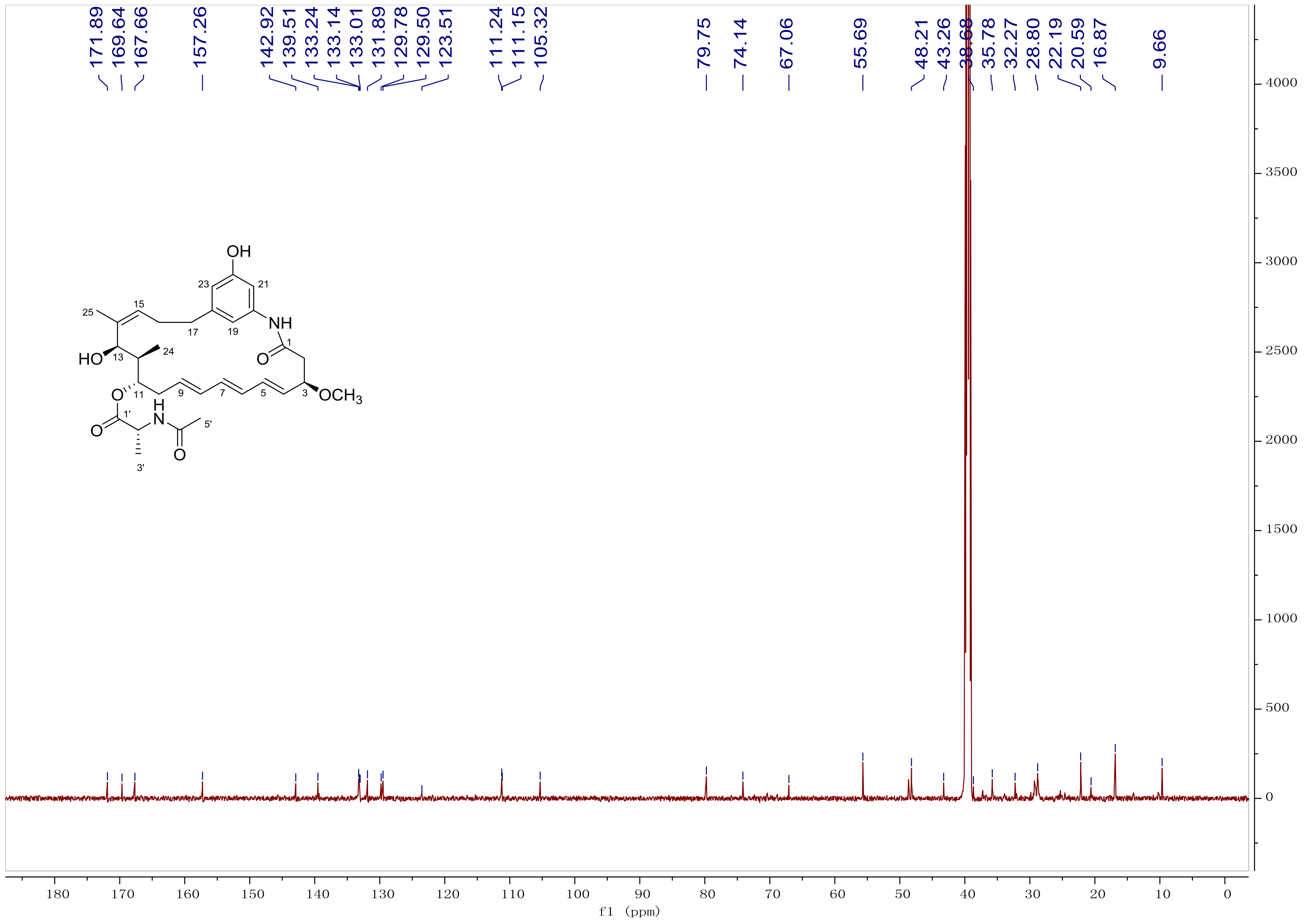

Figure S4. DEPT spectrum of trienomycin H (**1**) in DMSO-d_6

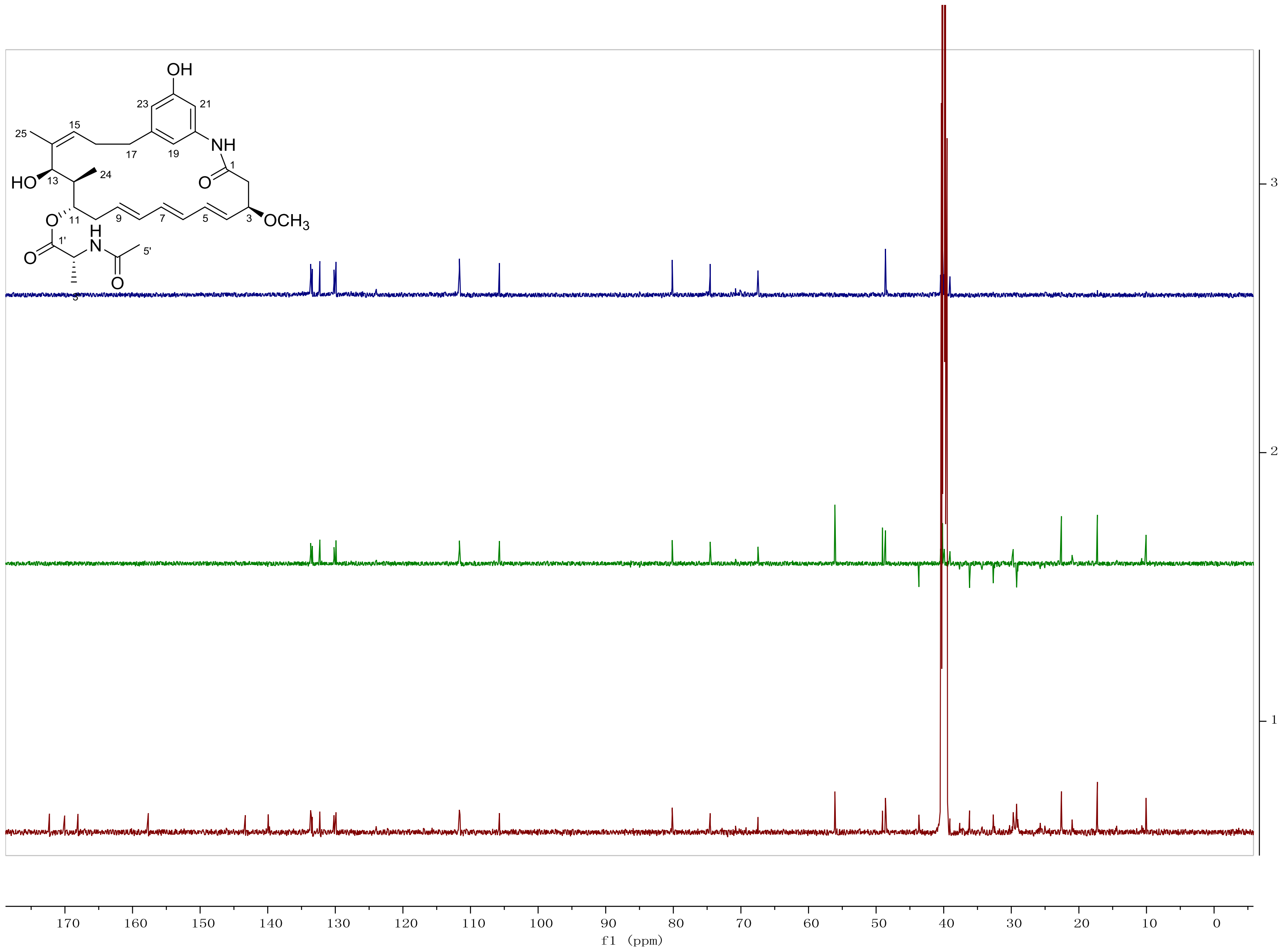

Figure S5. HSQC spectrum of trienomycin H (**1**) in DMSO-d_6

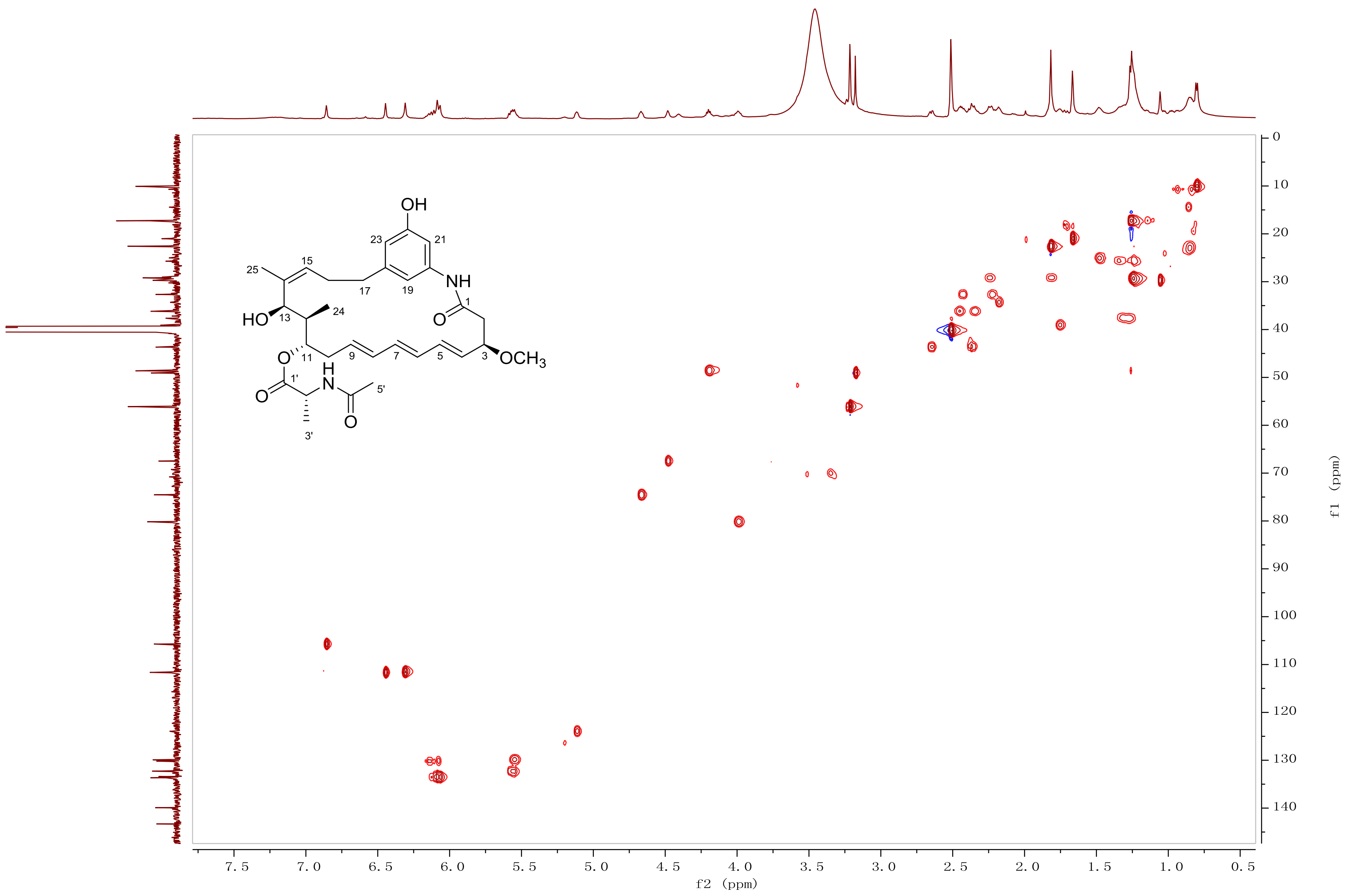

Figure S6. ^{1}H-^{1}H COSY spectrum of trienomycin H (**1**) in DMSO-d_6

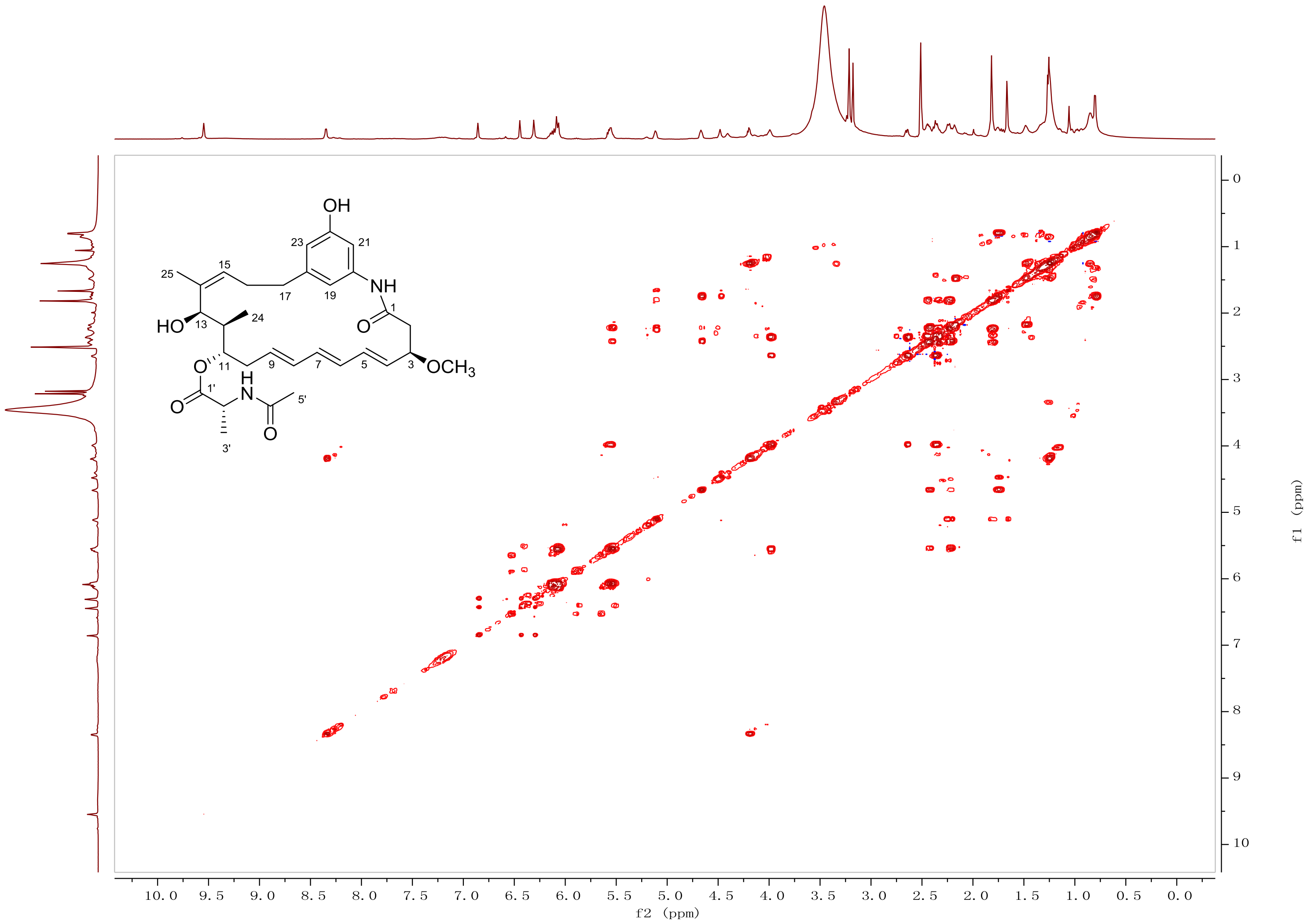

Figure S7. HMBC spectrum of trienomycin H (**1**) in DMSO-d_6

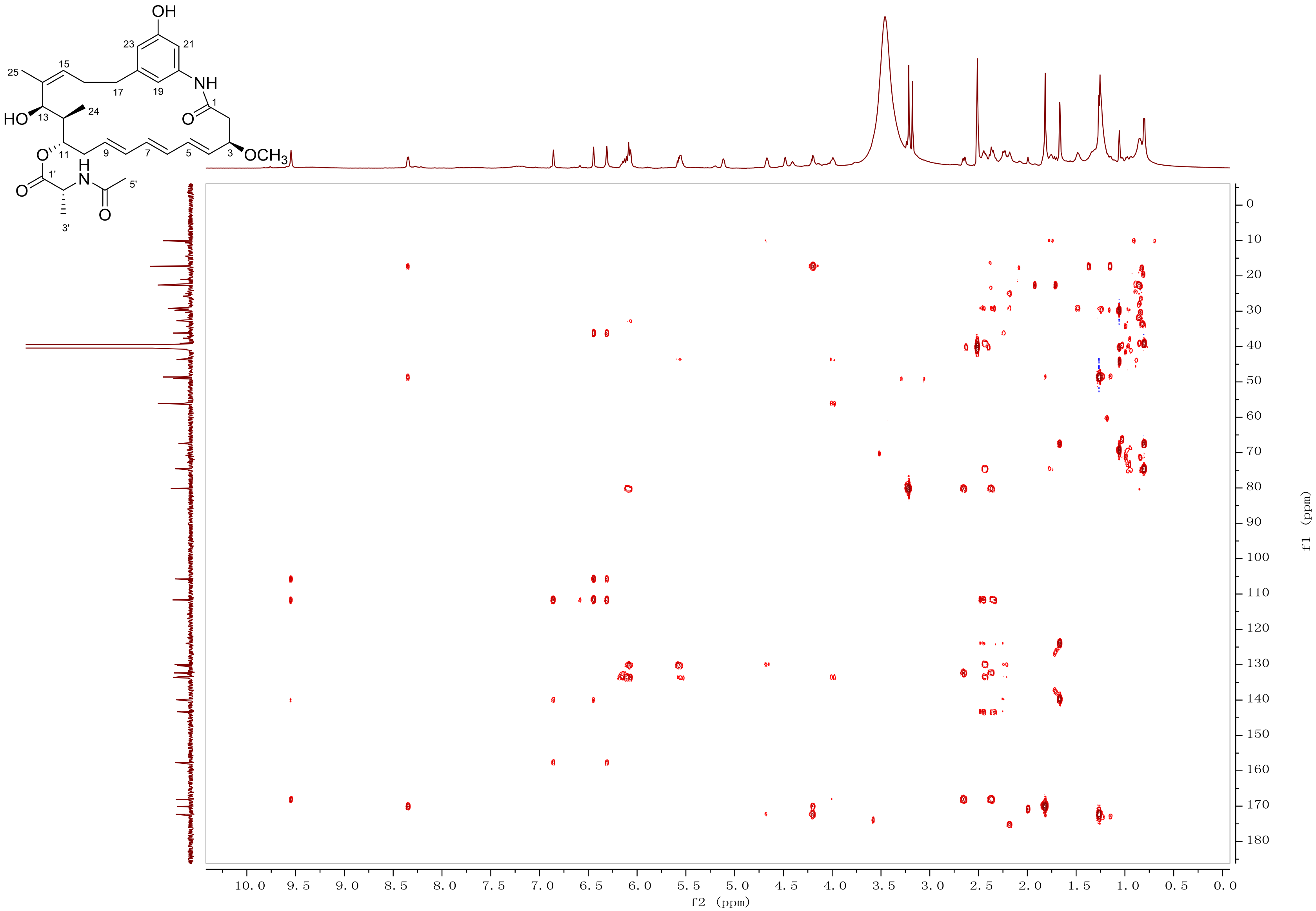

Figure S8. HRESIMS spectrum of trienomycin I (**2**)

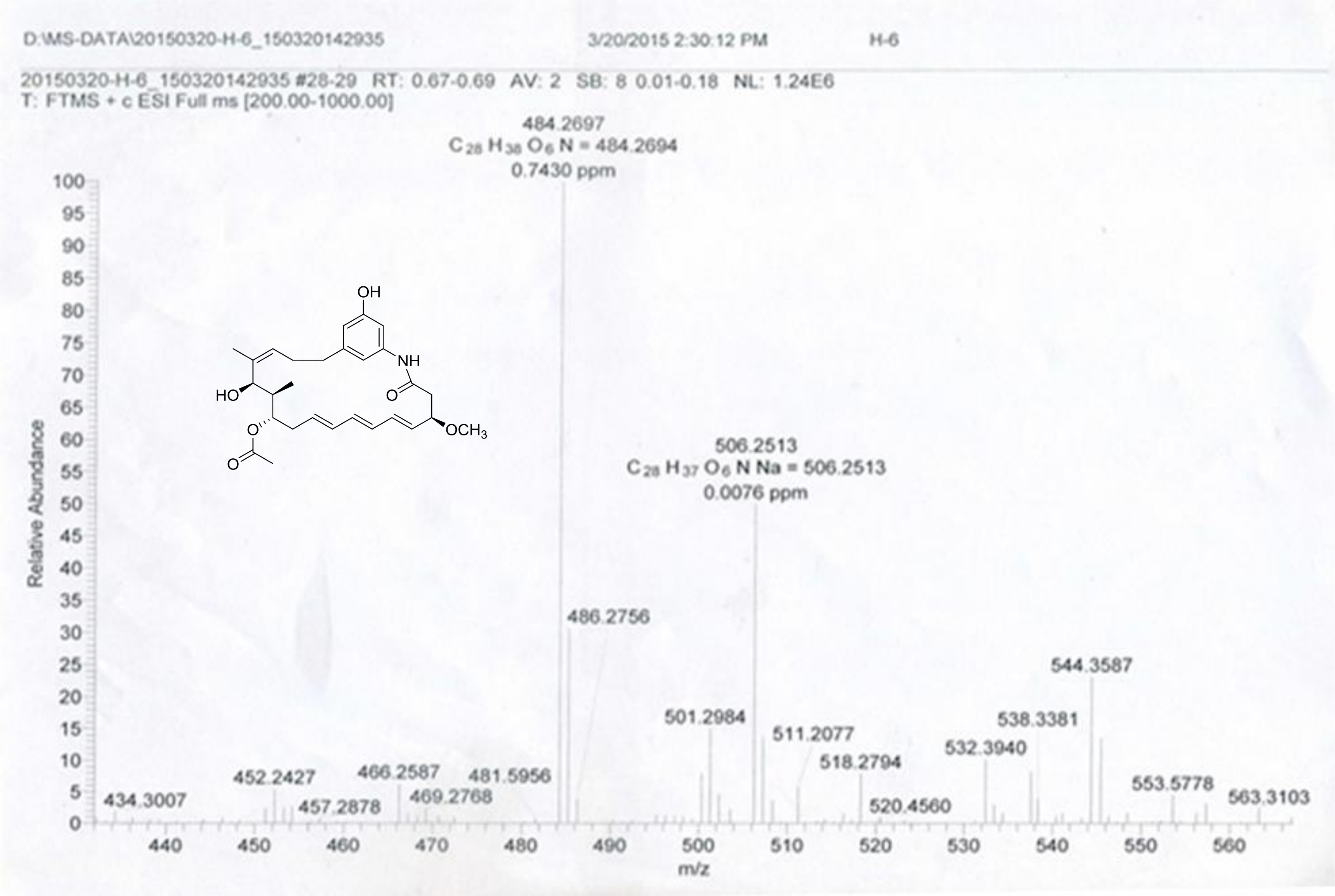

Figure S9. ^{1}H-NMR spectrum of trienomycin I (**2**) in DMSO-d_6

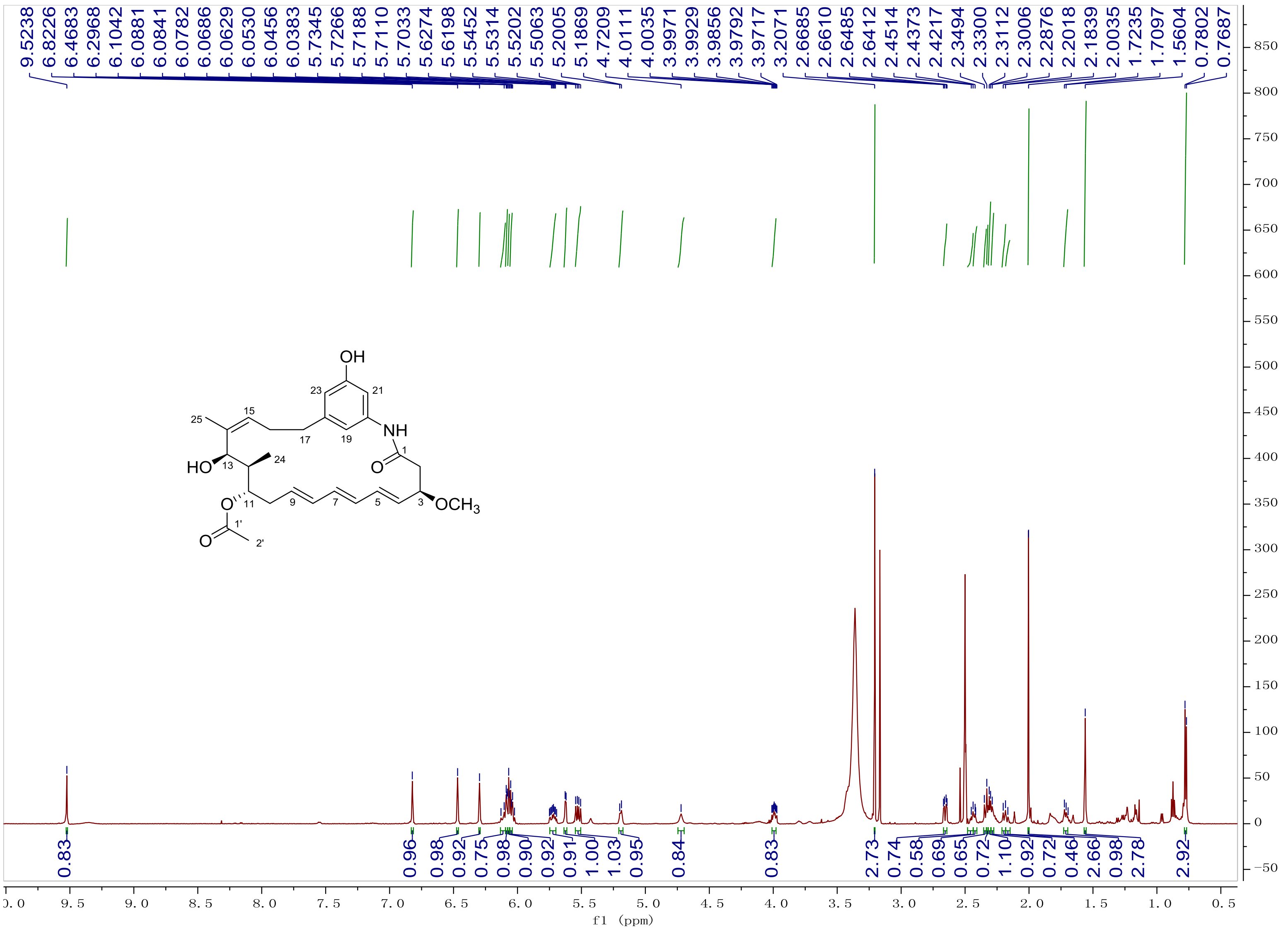

Figure S10. ^{13}C-DEPTQ-NMR spectrum of trienomycin I (**2**) in DMSO-d_6

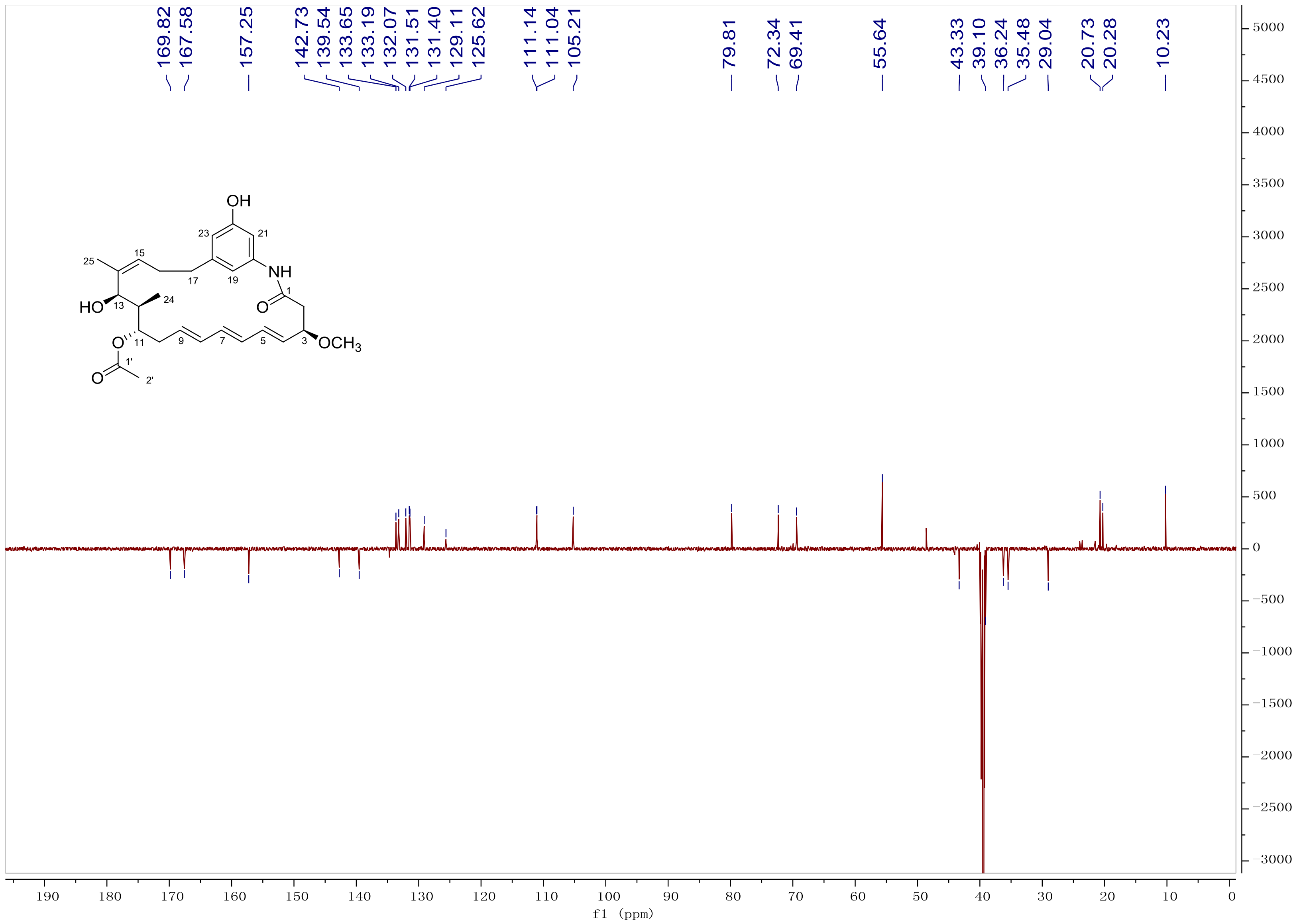

Figure S11. HSQC spectrum of trienomycin I (**2**) in DMSO-d_6

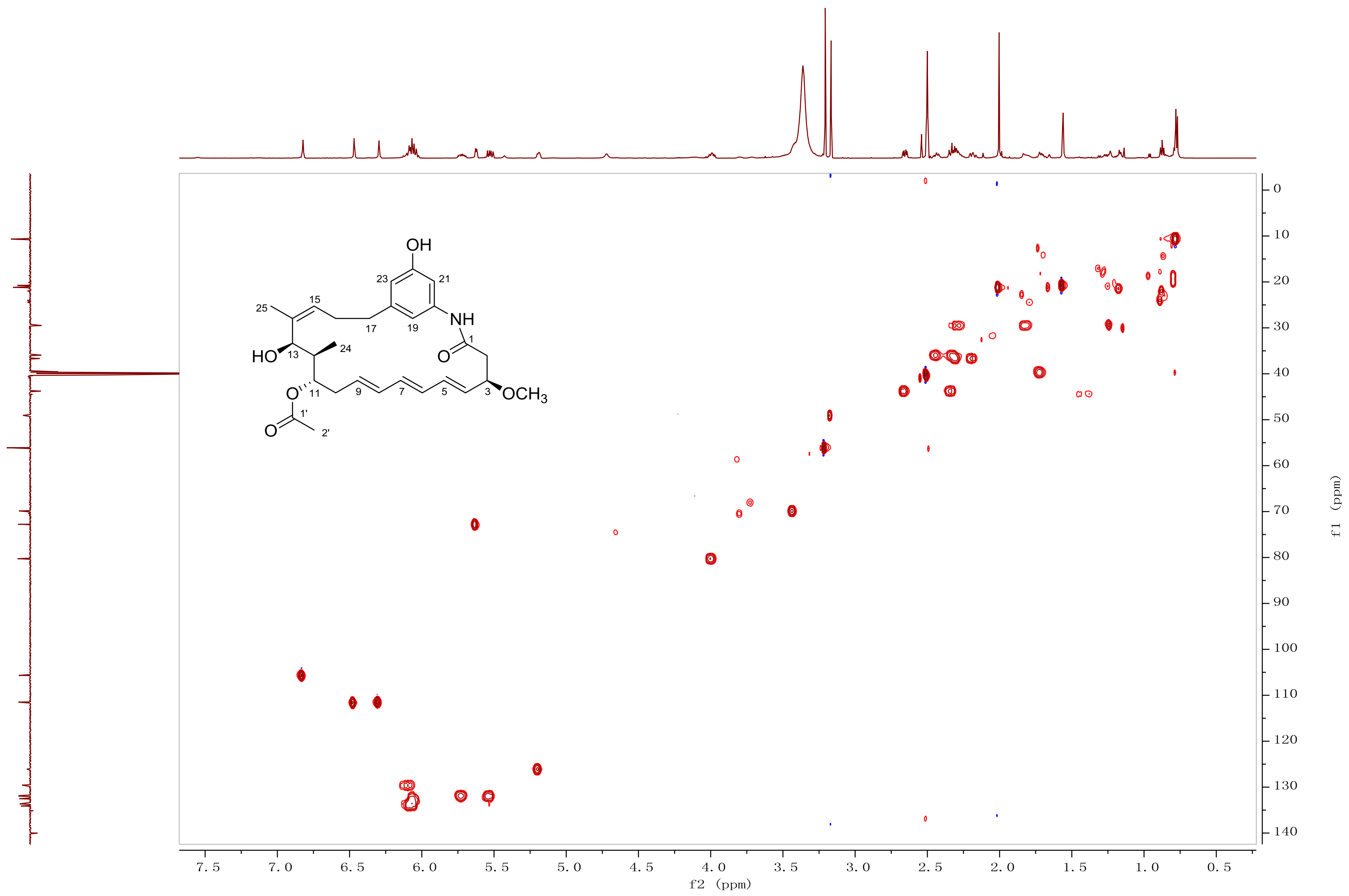

Figure S12. ^{1}H-^{1}H COSY spectrum of trienomycin I (**2**) in DMSO-d_6

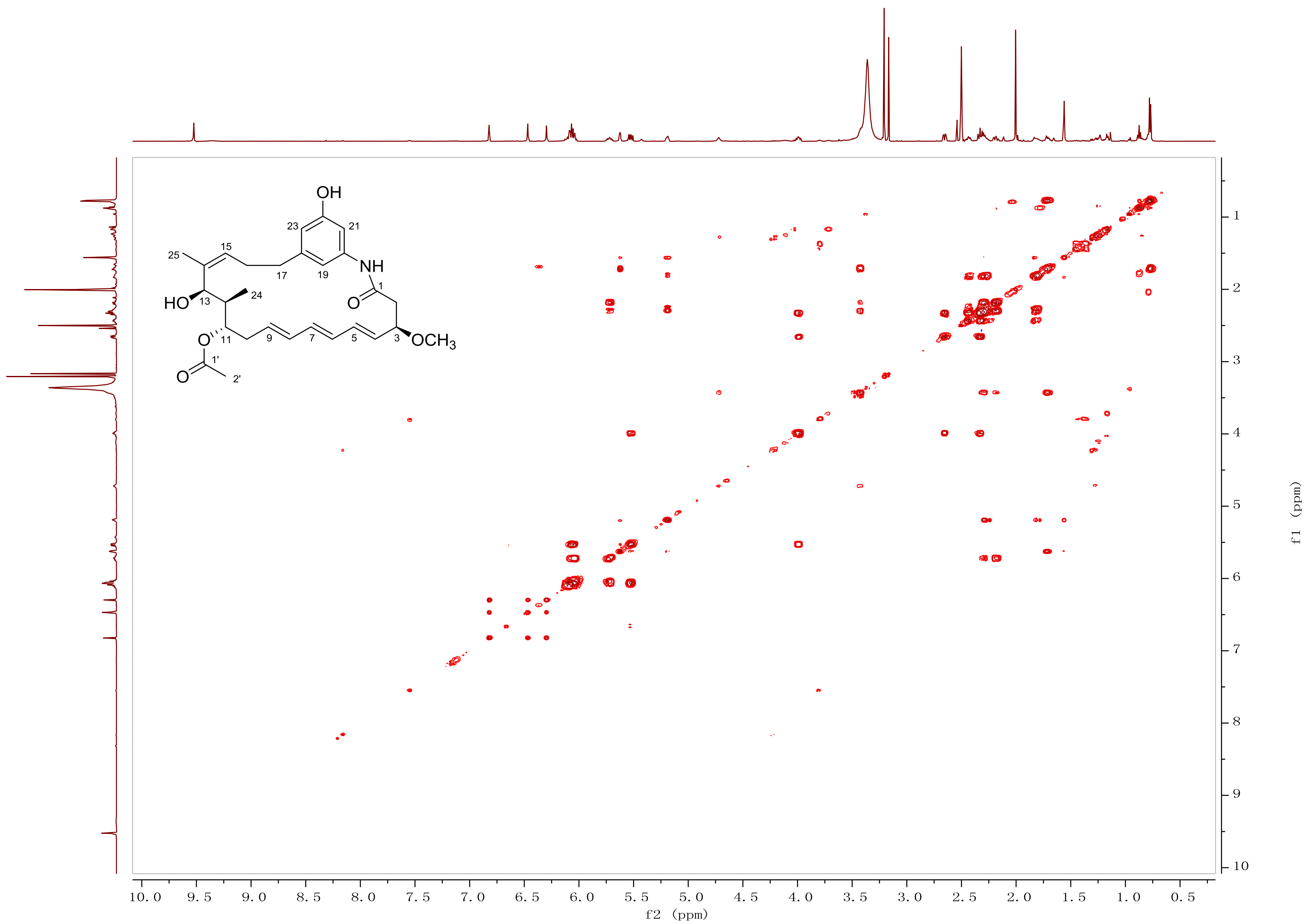

Figure S13. HMBC spectrum of trienomycin I (**2**) in DMSO-d_6

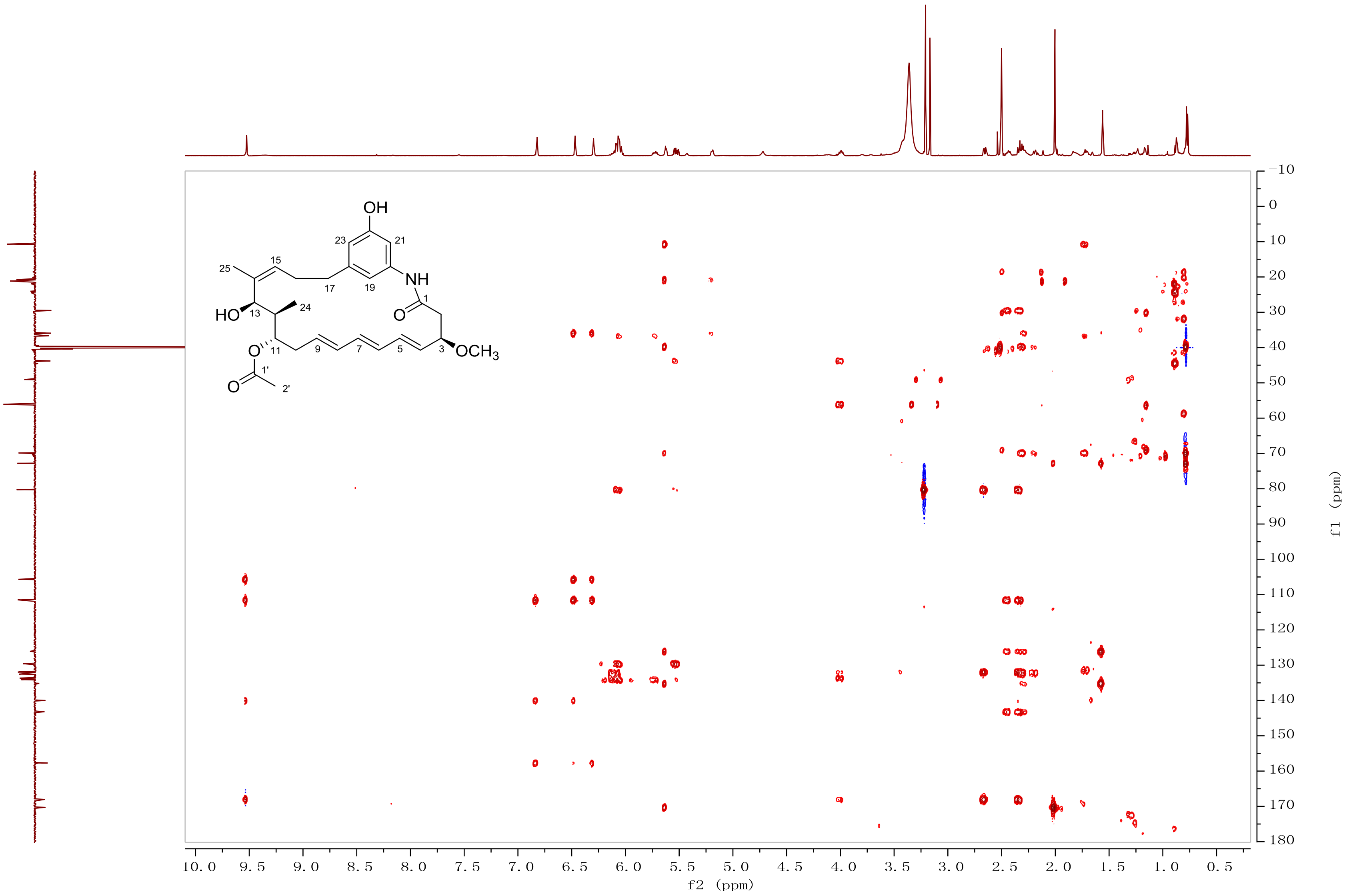

Figure S14. The determination of the Ala configuration of **1** by Marfey's Method (Solvents: A water + 0.2% TFA, B MeCN; linear gradient: 0 min, 25% B; 40 min, 60% B; 45 min, 100% B; temperature, 30 °C; flow rate, 1 mL/min; UV detection at λ 340 nm; FDAA, 14.2 min)

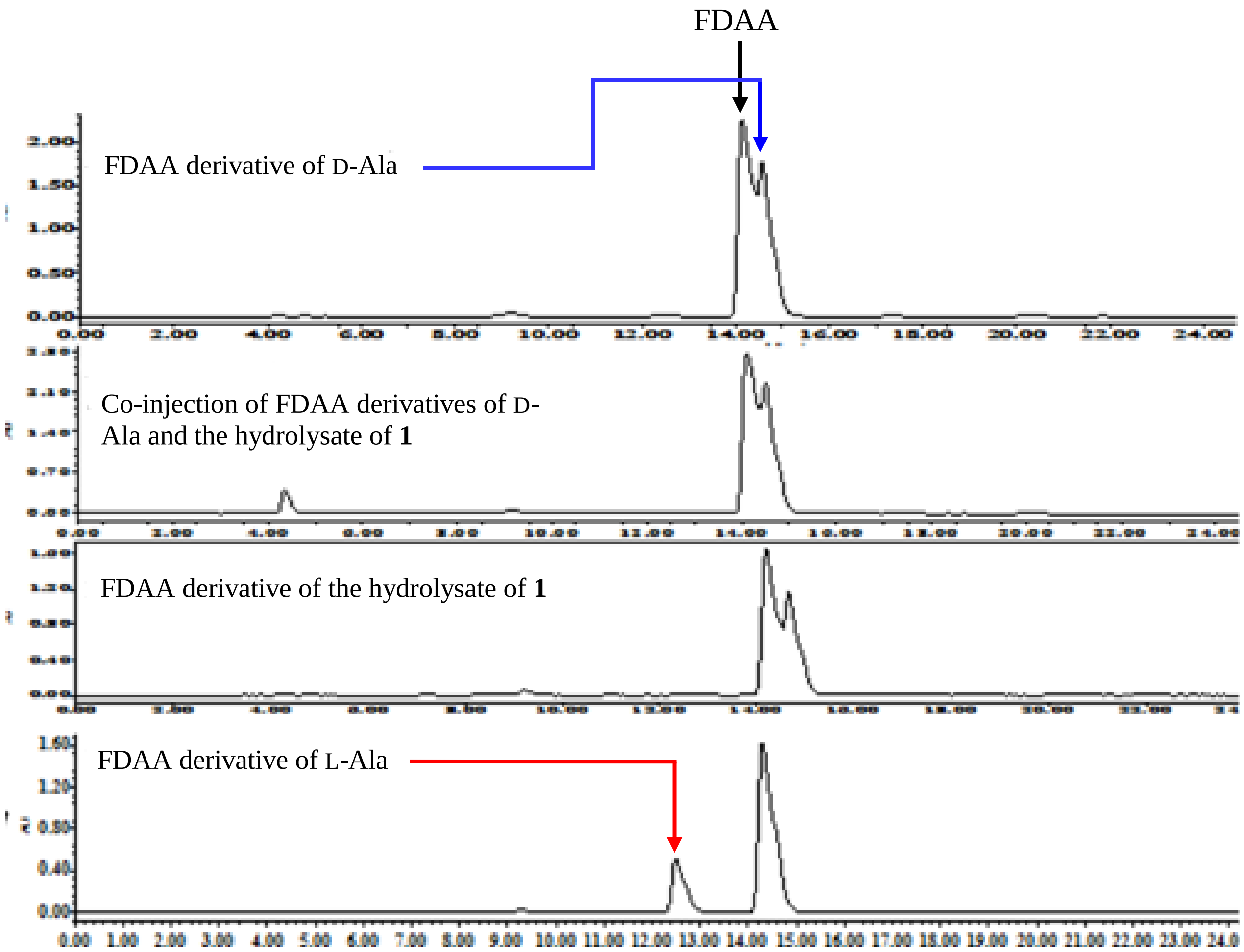

Figure S15. HPLC profiles for the water-insoluble hydrolysate of **1** and **3**

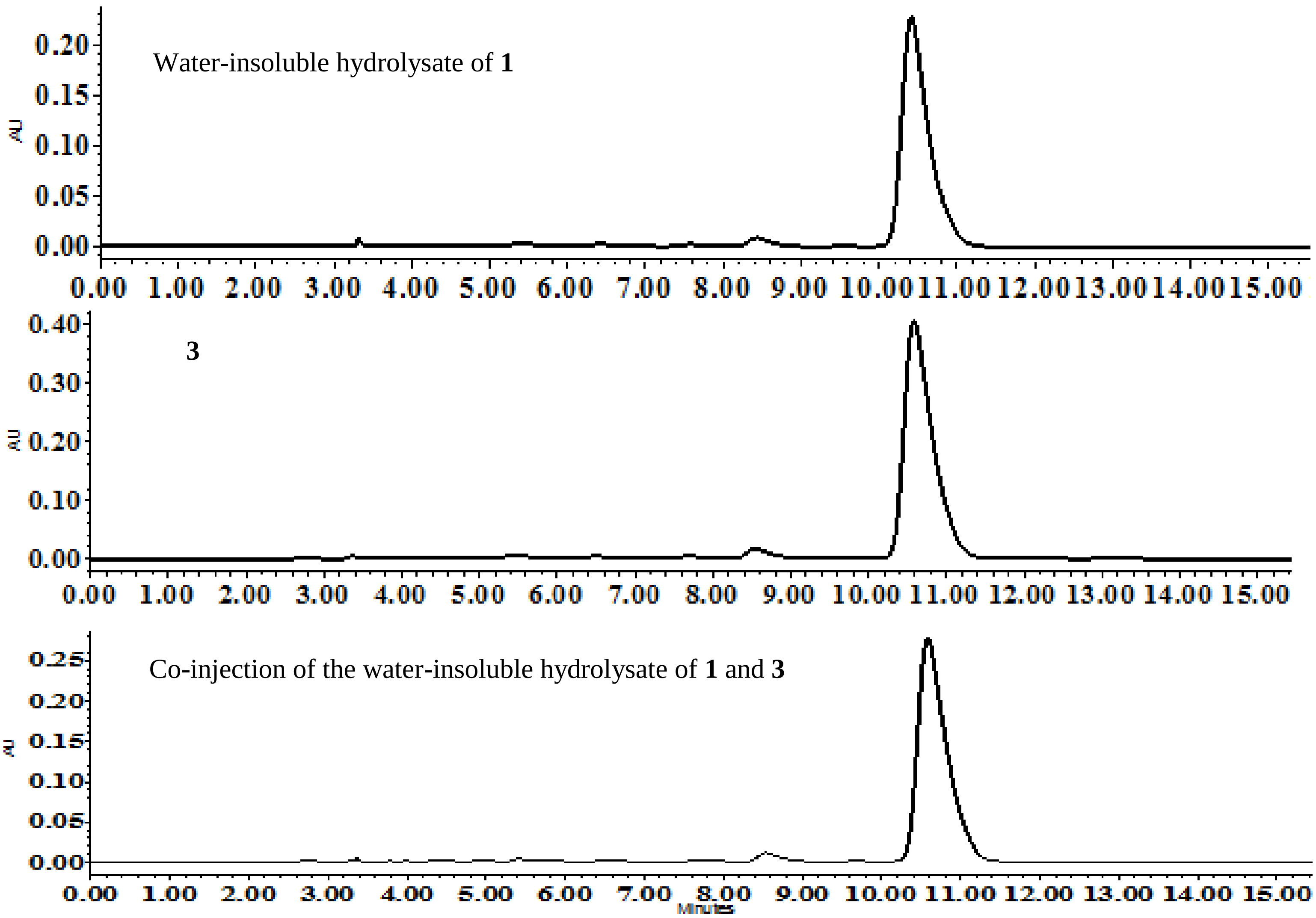

Figure S16. ESIMS spectrum for the water-insoluble hydrolysate of **1**

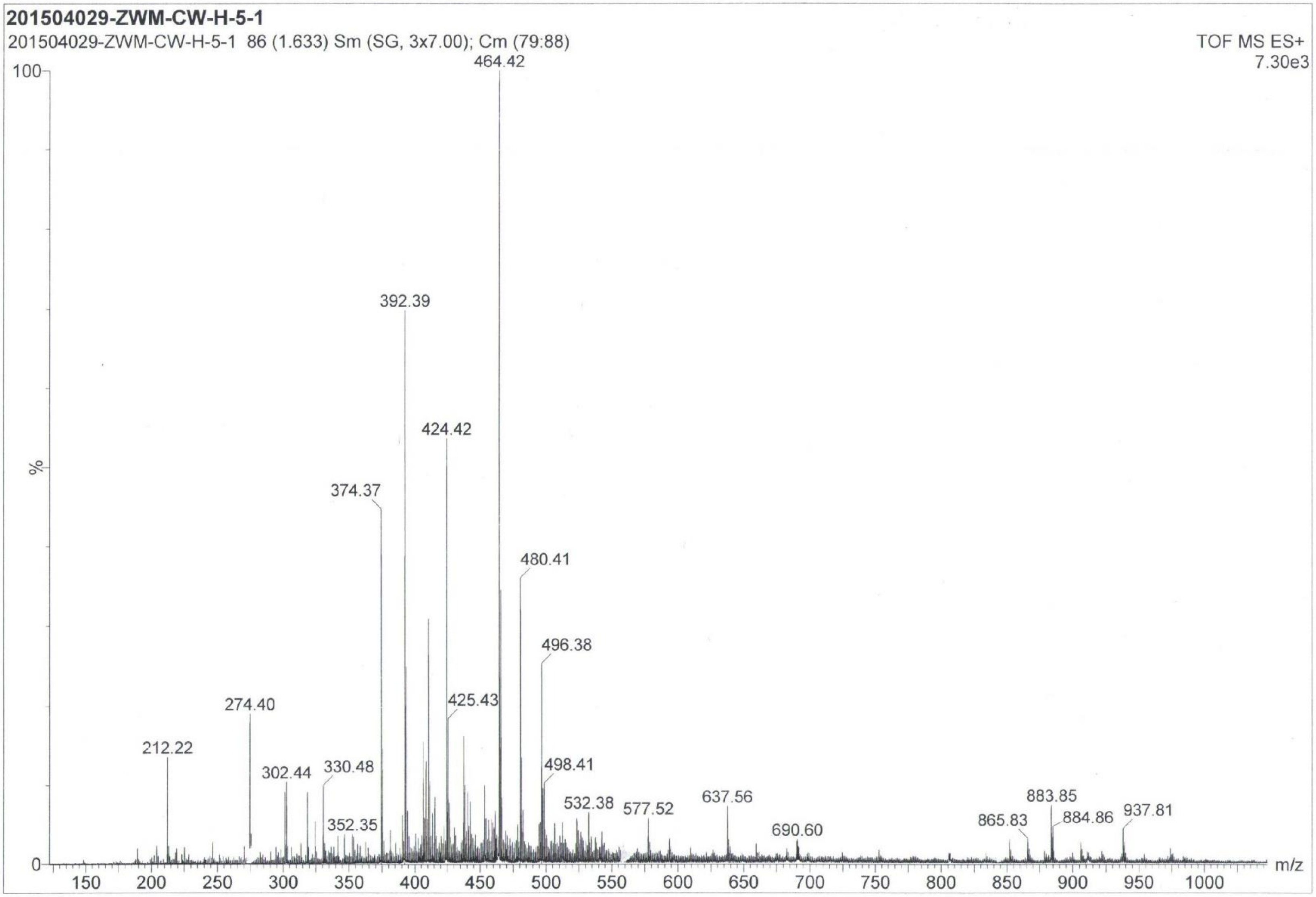

Figure S17. ESIMS spectrum for trienomycinol (**3**)

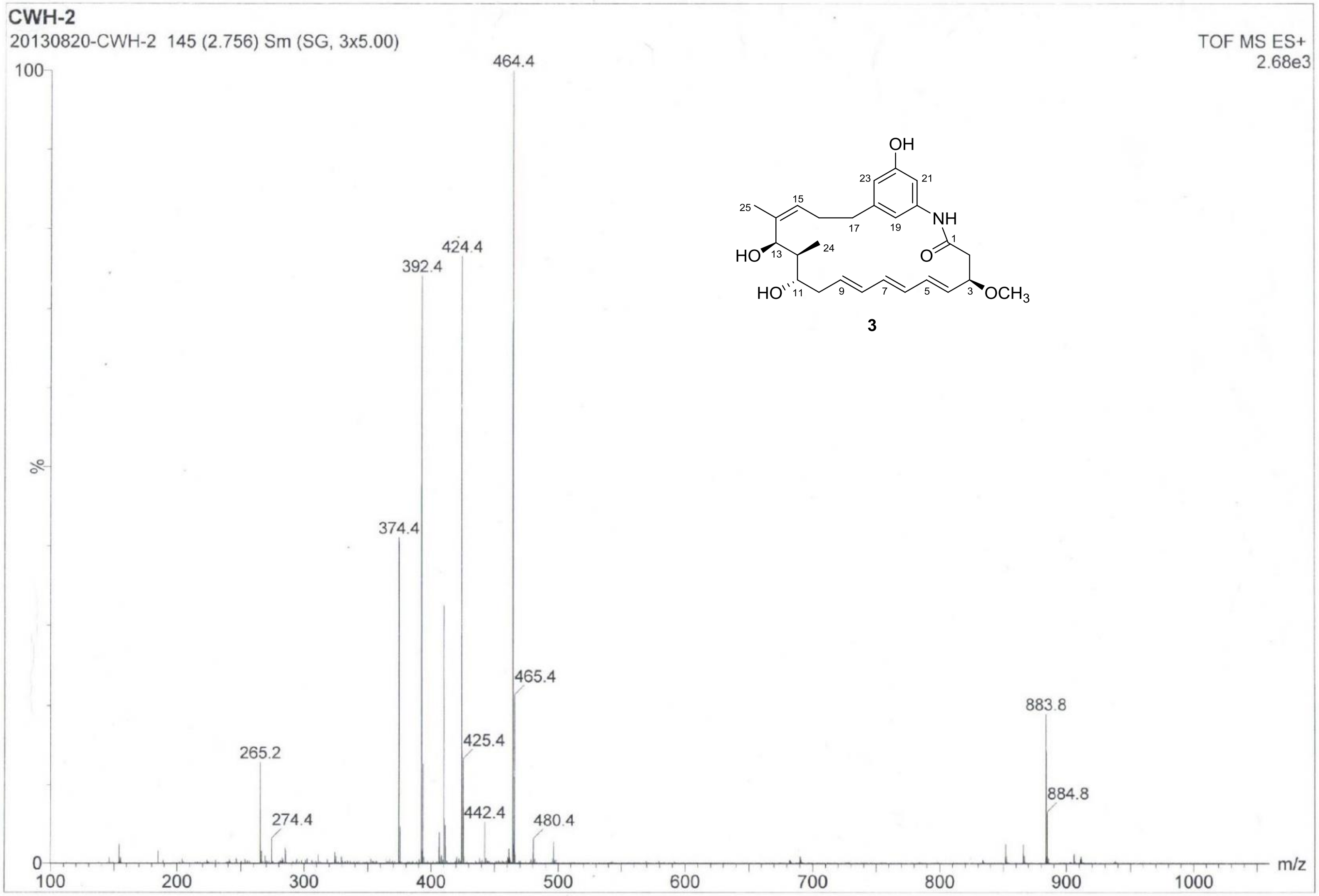

Figure S18. ^{1}H NMR spectra for **3** and the water-insoluble hydrolysate of **1** and **2**

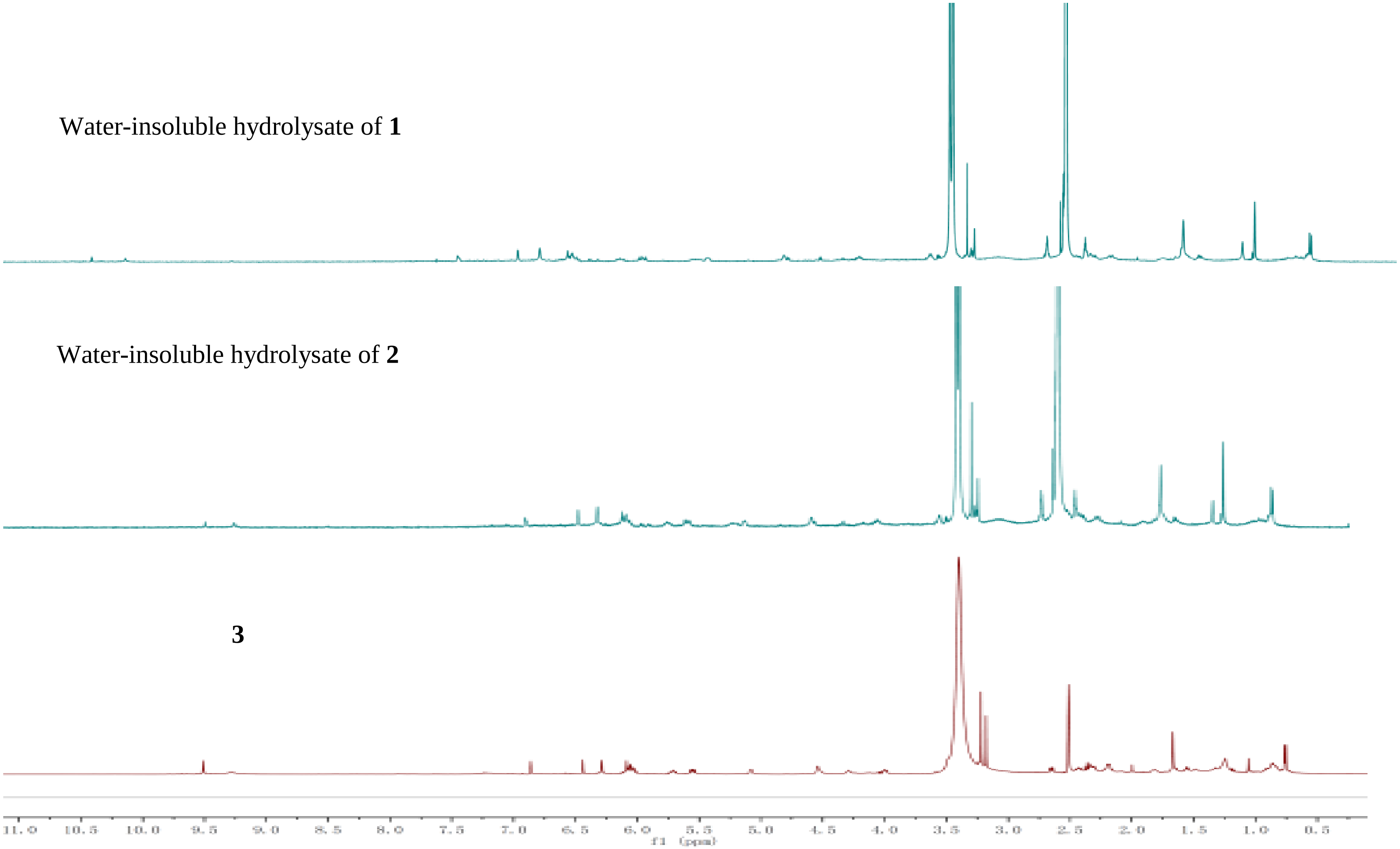

Figure S19. HPLC profiles for the water-insoluble hydrolysate of **2** and **3**

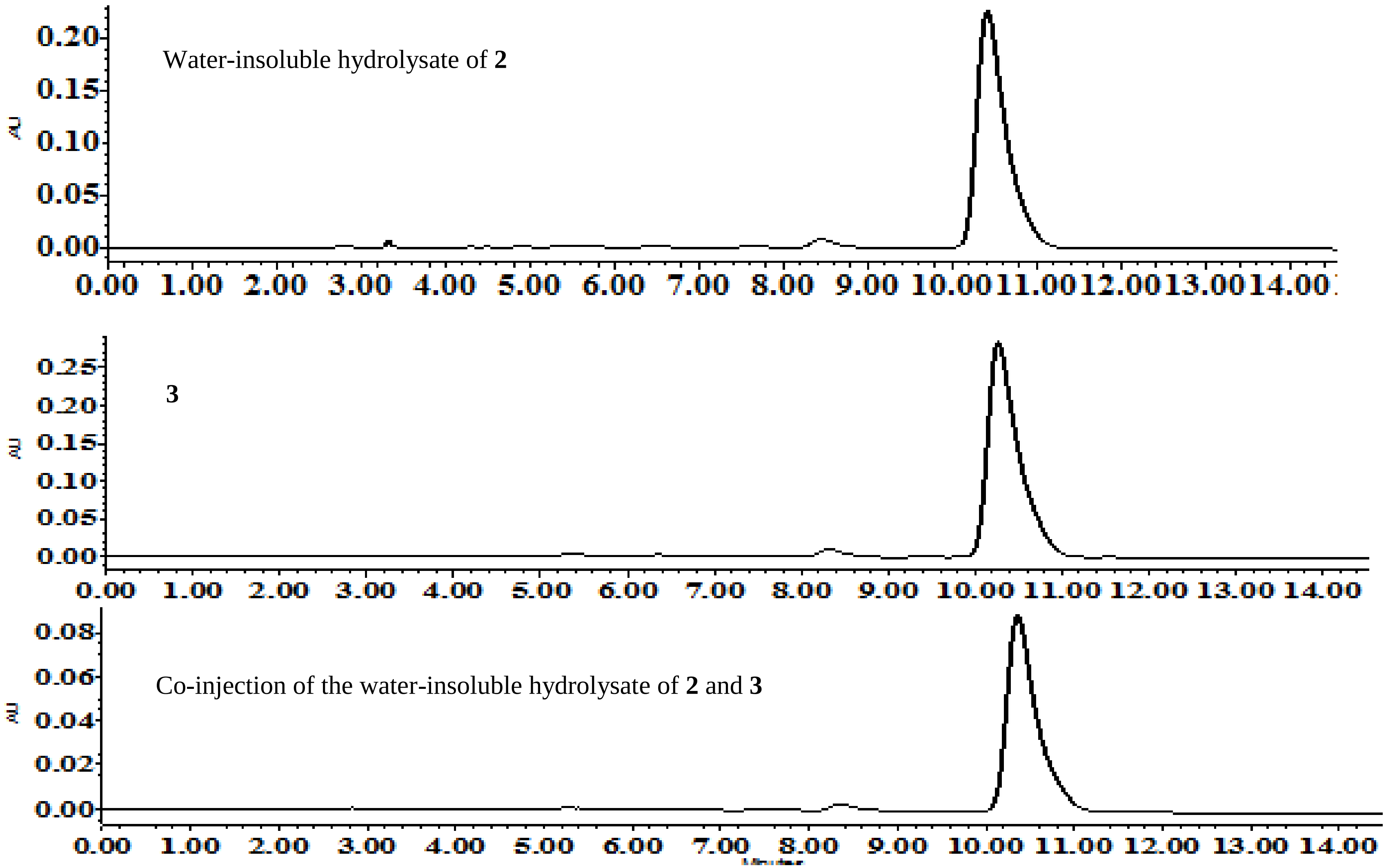

Figure S20. ESIMS spectrum for the water-insoluble hydrolysate of **2**

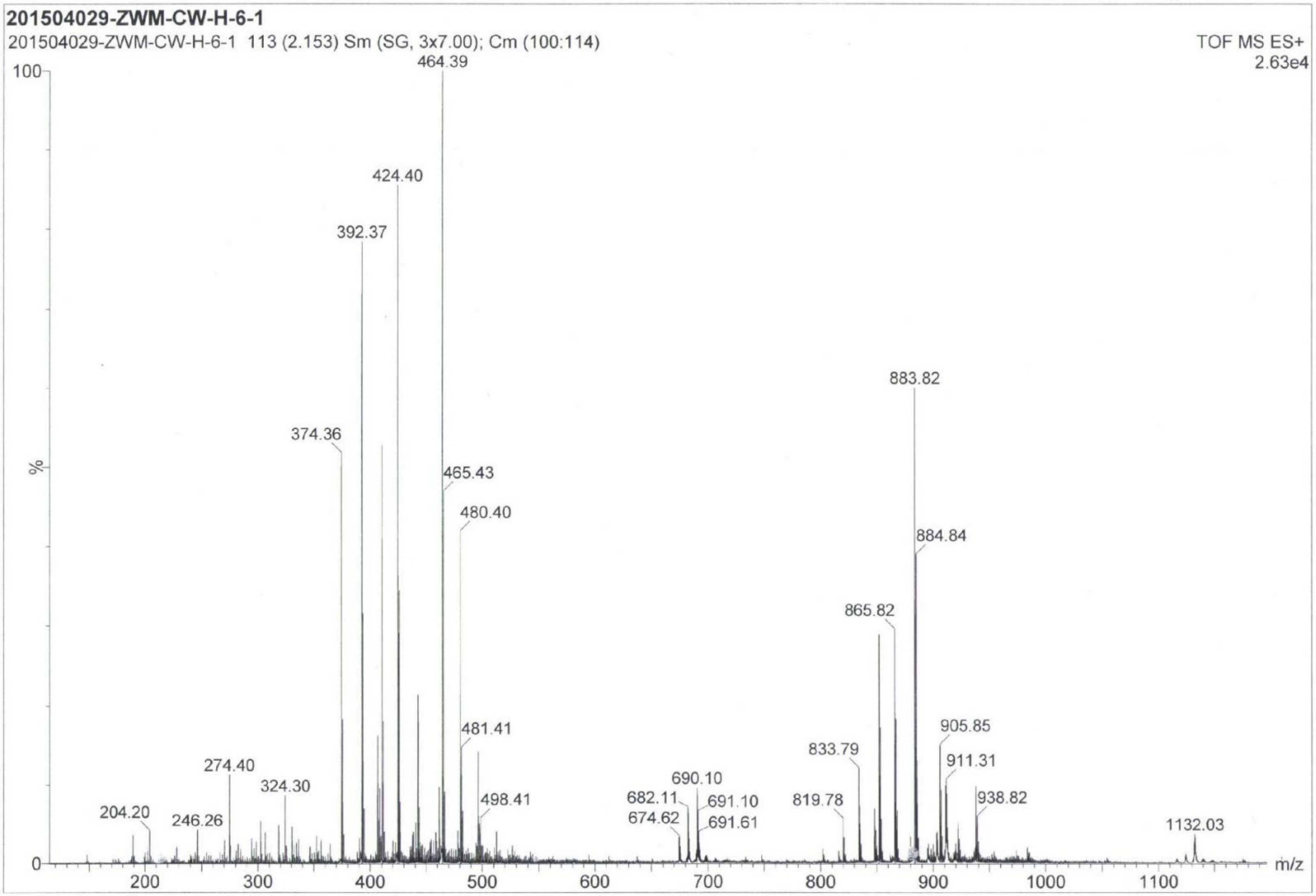

Figure S21. Phylogenetic tree mapping for the *Ochrobactrum* sp. OUCMDZ-2164

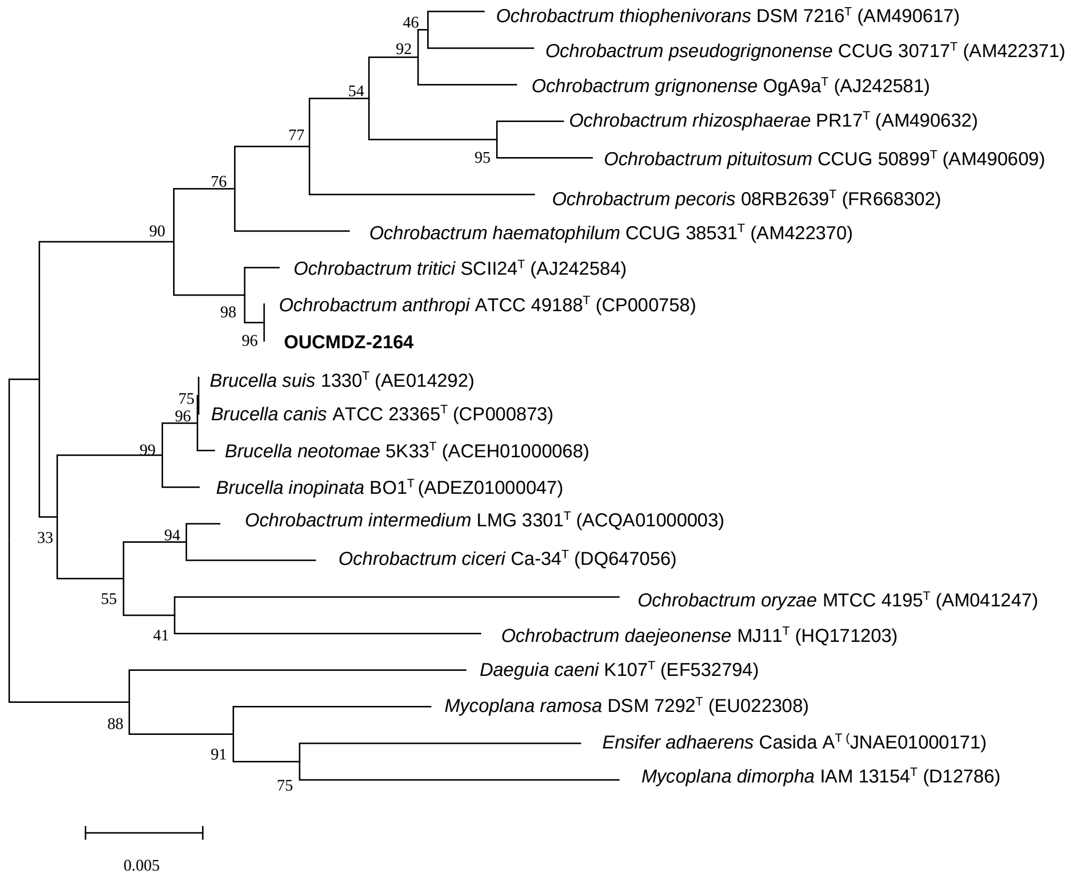

The Physical Properties of trienomycinol (3)

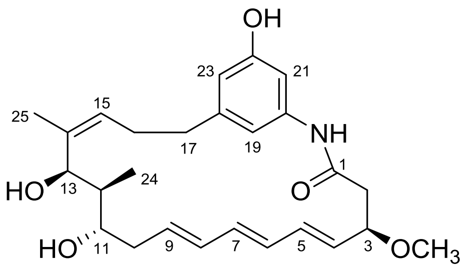

3

Trienomycinol (3): Yellow oil; $[\alpha]_D^{25}$ +62.8 (*c* 0.1, MeOH); ECD (0.0011 *M*, MeOH) λ_{max} ($\Delta\varepsilon$) 211 (−6.3), 266 (−2.8), 269 (+13.0) nm; ^{1}H NMR (DMSO-d_6, 600 MHz) δ 9.51, (s, 1H, 20-NH), 6.86 (s, 1H, H-21), 6.44 (s, 1H, H-23), 6.29 (s, 1H, H-19), 6.09 (dd, *J* = 15.3 Hz, *J* = 11.0 Hz, 1H, H-6), 6.08 (dd, *J* = 15.3 Hz, *J* = 10.3 Hz, 1H, H-7), 6.07 (dd, *J* = 15.0 Hz, *J* = 9.3 Hz, 1H, H-5), 6.06 (dd, *J* = 15.0 Hz, *J* =11.5 Hz, 1H, H-8), 5.71 (m, 1H, H-9), 5.56 (dd, *J* = 15.0 Hz, *J* = 8.3 Hz, 1H, H-4), 5.08 (m, 1H, H-15), 4.54 (brs, 1H, H-13), 4.00 (m, 1H, H-3), 3.48 (m, 1H, H-11), 3.18 (s, 3H, CH_3O-3), 2.65 (m, 1H, H_a-2), 2.42 (overlap, 1H, H_a-10), 2.37 (m, 1H, H_b-2), 2.33 (overlap, 1H, H_b-10), 2.19 (overlap, 1H, H_a-17), 2.18 (overlap, 1H, H_a-16), 2.00 (overlap, 1H, H_b-17), 1.82 (overlap, 1H, H_b-16), 1.67 (overlap, 1H, H-12), 1.67 (overlap, 3H, H_3-25), 0.75 (d, *J* = 6.6 Hz, 3H, H_3-24); ^{13}C NMR (DMSO-d_6, 150 MHz) δ 168.1 (C, C-1), 157.6 (C, C-22), 143.5 (C, C-18), 140.0 (C, C-14), 140.0 (C, C-20), 134.1 (CH, C-6), 133.5 (CH, C-5), 132.5 (CH, C-9), 132.1 (CH, C-8), 131.8 (CH, C-4), 129.4 (CH, C-7), 124.0 (CH_2, C-15), 111.5 (CH, C-19), 111.5 (CH, C-23), 105.7 (CH, C-21), 80.1 (CH, C-3), 70.7 (CH, C-11), 68.2 (CH, C-13), 56.1 (CH_3, CH_3O-3), 43.7 (CH_2, C-2), 41.5 (CH, C-12), 36.8 (CH_2, C-10) 36.2 (CH_2, C-17), 29.3 (CH_2, C-16), 20.9 (CH_3, C-25), 10.5 (CH_3, C-24). ESIMS *m/z* 464.4 $[M + Na]^+$.
